# Single Amino Acid Modifications for Controlling the Helicity of Peptide-Based Chiral Gold Nanoparticle Superstructures

**DOI:** 10.1021/jacs.3c00827

**Published:** 2023-03-13

**Authors:** Sydney C. Brooks, Ruitao Jin, Victoria C. Zerbach, Yuyu Zhang, Tiffany R. Walsh, Nathaniel L. Rosi

**Affiliations:** † Department of Chemistry, University of Pittsburgh, Pittsburgh, Pennsylvania 15260, United States; ‡ Department of Chemical and Petroleum Engineering, University of Pittsburgh, Pennsylvania 15260, United States; § Institute for Frontier Materials, Deakin University, Geelong, Victoria 3216, Australia

## Abstract

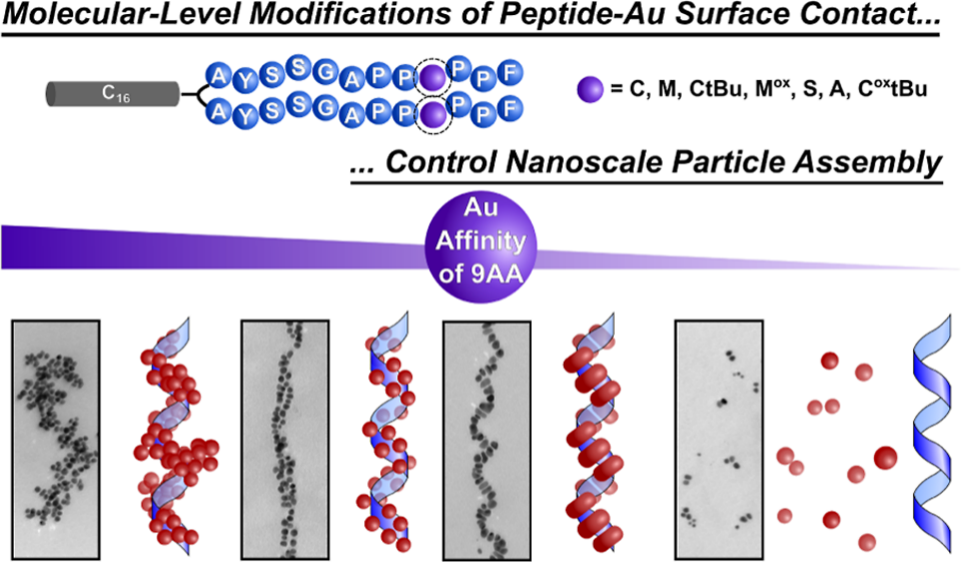

Assembling nanoparticles (NPs) into well-defined superstructures can lead to emergent collective properties that depend on their 3-D structural arrangement. Peptide conjugate molecules designed to both bind to NP surfaces and direct NP assembly have proven useful for constructing NP superstructures, and atomic- and molecular-level alterations to these conjugates have been shown to manifest in observable changes to nanoscale structure and properties. The divalent peptide conjugate, C_16_-(PEP_Au_)_2_ (PEP_Au_ = AYSSGAPPMPPF), directs the formation of one-dimensional helical Au NP superstructures. This study examines how variation of the ninth amino acid residue (M), which is known to be a key Au anchoring residue, affects the structure of the helical assemblies. A series of conjugates of differential Au binding affinities based on variation of the ninth residue were designed, and Replica Exchange with Solute Tempering (REST) Molecular Dynamics simulations of the peptides on an Au(111) surface were performed to determine the approximate surface contact and to assign a binding score for each new peptide. A helical structure transition from double helices to single helices is observed as the peptide binding affinity to the Au(111) surface decreases. Accompanying this distinct structural transition is the emergence of a plasmonic chiroptical signal. REST-MD simulations were also used to predict new peptide conjugate molecules that would preferentially direct the formation of single-helical AuNP superstructures. Significantly, these findings demonstrate how small modifications to peptide precursors can be leveraged to precisely direct inorganic NP structure and assembly at the nano- and microscale, further expanding and enriching the peptide-based molecular toolkit for controlling NP superstructure assembly and properties.

## Introduction

The compositions and structures of molecules are the foundation of complexity and diversity. This is particularly apparent in biology, where small differences in nucleic acid sequence (genotype) can dramatically influence observable characteristics (phenotype). Equally striking is how the inversion of a single chiral center within a small molecule can result in different properties and functions. Harnessing the precision of molecular structure and translating it across length scales to the “nano” regime can enable molecular-level coding of nanoscale structure and properties and nano-architecting approaches that rely on well-established methods for finely controlling the molecular structure.^1−3^

**Figure 1:**
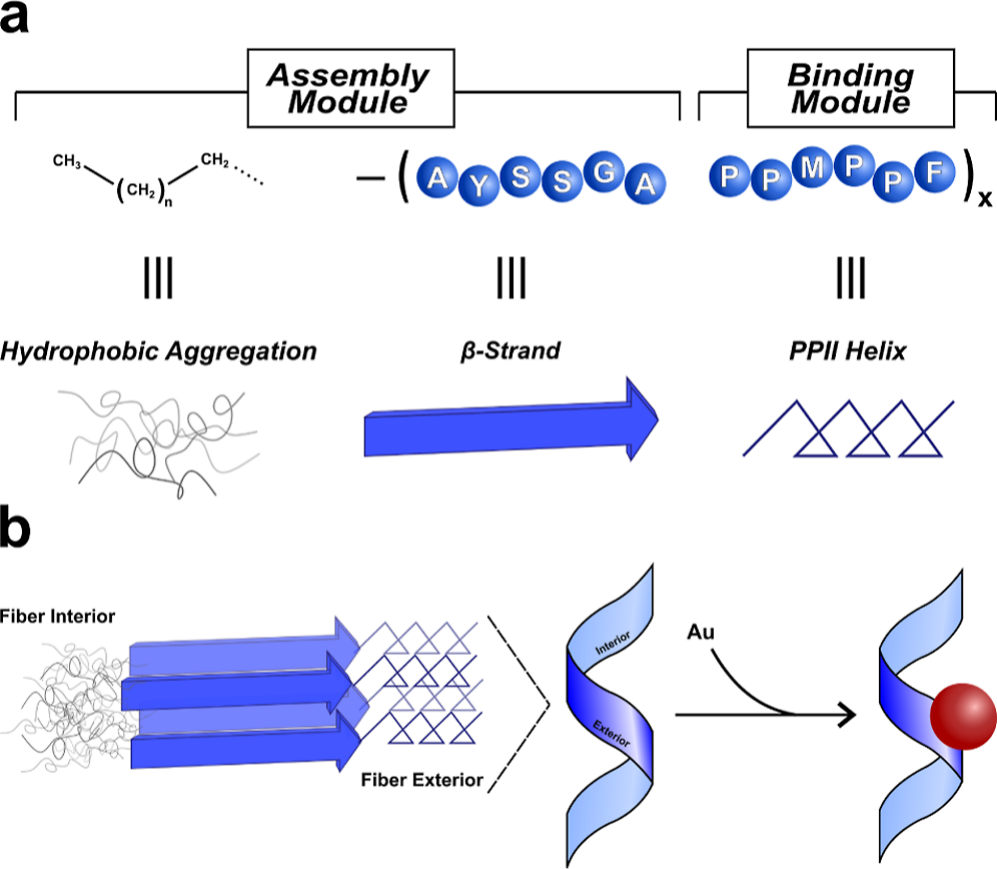
(a) Illustration of the different “modules” within the peptide conjugate: the assembly module contains the hydrophobic tail and β-strands at the peptide N-terminus; the C-terminus is the NP binding module and adopts PPII secondary structure. (b) Peptide conjugates can assemble into one-dimensional helical fibers with the NP binding module exposed to the aqueous environment and the assembly module sequestered in the interior of the fiber.

We and others investigate how peptides can be used as programmable molecular species for controlling the synthesis and structure of metal nanoparticles (NPs)^4−8^ as well as their assembly into well-defined NP superstructures.^9−12^ Short-peptide (∼8 to 12 amino acids) NP capping ligands, composed of both natural and non-natural amino acids, provide a vast sequence space that can be leveraged to control NP size, shape, and properties. In our own work, we use amphiphilic peptide conjugate molecules to assemble Au NP superstructures.^9,13,14^ We have developed robust peptide conjugate assembly models that serve as the foundation for building connections between the molecular composition, structure, and properties of NP superstructures.^15−18^ In general, the peptide conjugates contain a hydrophobic organic tail appended to the N-terminus of one or more Au binding peptides (AYSSGAPPMPPF; initially reported as A3,^4^ herein referred to as PEP_Au_). In the context of Au NP assemblies, these conjugates contain both an assembly module and a NP binding module (Figure 1).

The assembly module (Figure 1a) consists of the N-terminal amino acids (AYSSGA) and the hydrophobic organic tail; a combination of parallel β-sheet secondary structure formation and hydrophobic aggregation promotes assembly in aqueous media (Figure 1b). The C-terminus (PPMPPF) is the NP binding module (Figure 1a). Together, the composition of these modules is a molecular code that we can manipulate to design and program diverse collections of NP superstructures. Substantial variation of the code can result in entirely different structural outcomes. For example, C_6_-A_2_-PEP_Au_ directs assembly of spherical NP superstructures,^13,19^ while C_12_-PEP_Au_ yields Au NP double helices.^9^ Fine-tuning the assembly architecture can be accomplished by making more subtle changes: adjusting the aliphatic tail length by 2 methylene units enables incremental tuning of helical pitch,^14,16^ use of either *L* or *D* amino acids yields left- or right-handed NP helices, respectively,^20^ and altering the sequence of amino acids in the NP binding module allows control over NP dimensions.^17^ Accompanying each of these molecularly programmed structural modifications are measurable differences in collective plasmonic properties.^16,17,20^

At an even finer level, we found that atomic-level changes to the NP binding module can also influence the superstructure morphology. In 2015, we reported a family of divalent peptide conjugates (C_*x*_-(PEP_Au_)_2_, *x* = 16–18) that direct the formation of double-helical Au NP assemblies.^14^ Later, we discovered that these conjugates yield single-helical superstructures with oblong NPs when their methionine residues are oxidized from the thioether to the sulfoxide (i.e., C_*x*_-(PEP_Au_^M-ox^)_2_).^15^ The dramatic shift in structure upon oxidation of the methionine residues led to a strong plasmonic chiroptical response, indicating that small atomic modifications to the peptide conjugate molecular code could trigger significant property changes/enhancement.

Collectively, these observations prompted studies to uncover the origin of this structural phenomenon with the aims of (i) understanding how and why the NP binding module affects the NP superstructure morphology, (ii) identifying new peptide sequences that would exclusively direct formation of single-helical superstructures, and (iii) developing new insights into atomic/molecular factors that could influence the structure and properties of NP superstructures fabricated using our peptide-based methodology.

## Experimental Section

### General Methods and Materials

All chemicals were purchased from commercial sources and used without further purification. Peptides were synthesized using established microwave-assisted solid-phase peptide synthesis procedures using a CEM Mars microwave. For all aqueous solutions, NanoPure water (18.1 mΩ) from a Barnstead Diamond purification system was used. The peptides and peptide conjugates were purified using reverse phase high-performance liquid chromatography (HPLC) on an Agilent 1200 liquid chromatographic system equipped with a diode array, multiple-wavelength detectors, and a Zorbax-300SB C_18_ column. Peptide and peptide conjugate masses were determined using liquid chromatography mass spectrometry (LC–MS) on a Shimadzu LC–MS 2020 instrument. Ultraviolet–visible (UV–vis) spectra were collected using an Agilent 8453 UV–vis spectrometer with a quartz cuvette (10 mm path length).

### Synthesis

#### Peptide Synthesis

All peptides were synthesized using established microwave-assisted solid-phase peptide synthesis protocols. Briefly, 138.8 mg (0.25 mmol) of Fmoc-Phe-Novasyn TGA resin (Millipore catalog no. 8560340001) was transferred to a filtration manifold and swelled in *N*,*N*′-dimethylformamide (DMF) for about 30 min. To remove the Fmoc protecting group from the resin, 2 mL of 20% 4-methylpiperidine in DMF solution was added, and the vessel was microwaved with agitation. The deprotection method on the microwave consisted of a 1 min temperature ramp to 75 °C, followed by a 2 min hold. The deprotection solution was removed by filtration, and the resin was rinsed with approximately 3 mL of DMF for 30 s (3X). The solid Fmoc-protected amino acids (4 equiv, 0.125 mmol) were activated in a 0.1 M solution of O-(1*H*-6-chlorobenzotriazole-1-yl)-1,1,3,3-tetramethyluronium hexafluorophosphate in 1-methyl-2-pyrrolidinone (5 equiv, 1.25 mL) and *N*,*N*-diisopropylethylamine (7 equiv, 0.175 mmol, 30.4 μL); they were vortexed to dissolve and then allowed to sit on the benchtop for at least 5 min. The activated amino acid solution was added to the resin vessel and microwaved with agitation using the following coupling method: 1 min temperature ramp to 75 °C, followed by a 5 min hold. The excess solution was drained, and the resin was again washed with DMF. This procedure was repeated for each subsequent amino acid. Every proline and proline-adjacent amino acid was double-coupled (i.e., coupling steps of 2 equivalents per amino acid were performed in sequence). The final step was either deprotection to produce amine-terminated peptides or deprotection and coupling of a 5-azido pentanoic acid cap, using the previously described coupling protocol.^14,15^ The completed sequence was cleaved from the resin with a mixture of 90% trifluoroacetic acid, 5% diisopropylsilane, and 5% NanoPure water. The product peptide was isolated by precipitation with cold diethyl ether, then lyophilized, and purified via HPLC. For the sequences that contained an oxidized residue (M^ox^ and C^ox^tBu), the lyophilized peptide was dissolved in 1 mL of 1:1 acetonitrile and NanoPure water with 8 μL of 50% hydrogen peroxide in NanoPure water. The solution was left undisturbed on the benchtop overnight, and then the oxidized peptide was collected via HPLC.

#### Peptide Conjugate Synthesis

The azide-terminated peptides were coupled to C_14_-dialkyne using established protocols described previously.^14,15^

### Assembly Protocols

All assembly experiments were performed at room temperature.

#### Peptide Conjugate Assembly

The lyophilized peptide conjugate (18.725 nmol) was dissolved in 250 μL of 0.1 M *N*-(2-hydroxyethyl)piperazine-*N*′-ethanesulfonic acid (HEPES) buffer. The solution was sonicated for 5 min, and then 2.5 μL of 0.1 M calcium chloride (CaCl_2_) was added to promote fiber assembly.

#### NP Superstructure Assembly

The lyophilized peptide conjugate (18.725 nmol) was dissolved in 250 μL of 0.1 M HEPES buffer. The solution was sonicated for 5 min, then 2.5 μL of 0.1 M calcium chloride (CaCl_2_) was added, and the solution was incubated on the benchtop for 25 min. Next, 2 μL of a 1:1 mixture of 0.1 M chloroauric acid (HAuCl_4_) in NanoPure water and 0.1 M triethylammonium buffer was added to the solution. When a black precipitate was observed, the solution was vortexed until the precipitate dissolved. The solution was incubated on the benchtop for ∼16 h to allow for complete superstructure growth.

### Characterization and Sample Preparation

#### Atomic Force Microscopy

Atomic force microscopy (AFM) images were collected on a Veeco MultiMode AFM with NanoScope V Controller in the tapping mode. The 0.1% 3-Aminopropyl-triethoxy-silane solution was drop-cast onto a freshly cut mica surface, rinsed with NanoPure water, and allowed to dry in a desiccator overnight. 50 μL of the peptide conjugate in 0.1 M HEPES (75 μM) was then drop-cast and rinsed with water after 10 min and allowed to dry.

#### Circular Dichroism Spectroscopy

The lyophilized peptide conjugate (18.725 nmol) was dissolved in 250 μL of 0.01 M HEPES buffer with 2.5 μL of CaCl_2_ and allowed to incubate overnight. CD measurements were collected using an Olis DSM 17 CD spectrometer with a quartz cuvette (0.1 cm path length) at 25 °C with a scan rate of 8 nm/min. For CD spectra of the Au NP assemblies, structures were prepared according to the synthetic protocol, and spectra were collected using the same instrument settings.

#### Attenuated Total Reflectance Fourier Transform Infrared Spectroscopy

Spectra were recorded on a PerkinElmer Spectrum 100 FTIR instrument equipped with an attenuated total reflectance (ATR) accessory using PerkinElmer Spectrum Express software. The lyophilized peptide conjugate (18.725 nmol) was dissolved in 250 μL of 0.1 M HEPES buffer with 2.5 μL of CaCl_2_ and incubated at room temperature overnight. 175 μL of the solution was dialyzed against NanoPure water using d-tube dialyzers (Millipore catalog no. 71505-3) and then concentrated by evaporation. 1 μL of the concentrated solution was drop-cast onto the ATR surface and allowed to dry before spectra were recorded.

#### Transmission Electron Microscopy

Low-magnification transmission electron microscopy (TEM) images were collected on a FEI Morgagni 268 instrument operated at 80 kV and equipped with an AMT side mount charge-coupled device camera system, and high-magnification TEM images were collected on a Hitachi H-9500 microscope operating at 100 kV (for peptide conjugate fibers) or 300 kV (for Au NP assemblies). TEM samples were prepared on a 3 mm-diameter copper grid with the Formvar coating according to the previously described protocol.^14,15^ Images were analyzed using ImageJ software.

### Molecular Simulations

#### Replica Exchange with Solute Tempering Molecular Dynamics Simulations

All simulations were performed using the GROMACS software package (version 2021).^21^ The simulation system comprised one Au(111) slab placed in an orthorhombic periodic simulation cell of dimensions 5.8 × 6.1 × 6.8 nm, with the *z*-axis perpendicular to the Au(111) plane. All simulations were performed in the Canonical (*NVT*) ensemble at 300 K, using the Nose–Hoover thermostat.^22,23^ The CHARMM22* force field^24,25^ was used to provide parameters for the peptides, the modified TIP3P model^26^ was used for water, and the GolP-CHARMM force-field^10^ used for the Au–peptide interactions. Full details are provided in the Supporting Information.

Replica exchange with solute tempering molecular dynamics (REST-MD) simulations for each of the six peptides (PEP_Au_^A,9^, PEP_Au_^S,9^, PEP_Au_^C,9^, PEP_Au_^T,9^, PEP_Au_^C(tBu),9^, and PEP_Au_^Cox(tBu),9^) were run in the adsorbed state at the aqueous Au(111) interface. Sixteen replicas were used with the “effective temperature” window of 300–430 K with Terakawa implementation.^27^ Before production REST-MD simulation, the 16 initial configurations were energy-minimized and then equilibrated at their target potential for 0.5 ns, with no exchange moves attempted during this period. REST-MD trajectories were of 15 ns duration (amounting to 16 × 15 ns = 0.24 μs of nominal total simulation time). The initial peptide backbone structures of the 16 replicas were taken from our previous runs.^17^ The 16 values of lambda used to scale our force field were λ_i_ = 0.000, 0.057, 0.114, 0.177, 0.240, 0.310, 0.382, 0.458, 0.528, 0.597, 0.692, 0.750,0.803, 0.855, 0.930, and 1.000.

#### Clustering Analysis

Clustering of all 15001 frames of each REST-MD simulation was performed over all backbone atoms using the Daura algorithm^28^ using the gmx-cluster utility with a cut-off of 2.0 Å in the root-mean-squared deviation (rmsd) of backbone atomic positions. The cross-cluster similarity was evaluated based on the rmsd of the backbone atoms of the relevant cluster centroid structures. A matched pair of clusters had a rmsd value 2.0 Å or less and a near-matched pair had a rmsd value less than 2.5 Å.

#### Residue Contact Analysis

To quantify the residue–surface contact for each residue in each peptide as predicted from the REST-MD simulations, the distance between the topmost Au layer and each residue was calculated. The residue was considered as in contact with the Au surface if the measured distance is equal to or less than the cut-off values which have been published elsewhere,^29^ along with the corresponding reference site for each residue. For non-standard residues, we used the sulfur atom as the reference site and a cut-off value of 4.5 Å for determining the surface contact. The summary of reference sites and cut-off values is provided in Table S2.

#### Steered MD and Umbrella Sampling Calculations

The umbrella sampling approach was used to evaluate the potential of mean force profiles for the amino acid analogues of the M^ox^, CtBu, and C^ox^tBu residues, binding at the aqueous Au(111) interface. The amino acid binding energy profiles were calculated using a methodology similar to that published previously.^30^ Both the N- and C-termini of the amino acids were capped. Steered pulling simulations were conducted to obtain configurations as a function of vertical distance from the surface in the *z*-direction. These were done with a constant speed, with a harmonic force constant for the steered MD (and for the subsequent umbrella sampling simulations) was 3000 kJ mol^–1^ nm^–2^, with a pulling rate of 0.05 nm ns^–1^. The spatial interval between adjacent umbrella sampling windows was 0.05 nm along the *z*-axis, and each umbrella sampling window was centered at each value of the reaction coordinate. For each window, an *NVT* simulation under the applied force constant was run for 100 ns. The resultant PMF profiles with estimated errors were obtained using the WHAM using the “traj” bootstrapping method with 200 bootstraps and a default tolerance of 10^–6^ in gmx-wham program.^31^

## Results and Discussion

**Figure 2:**
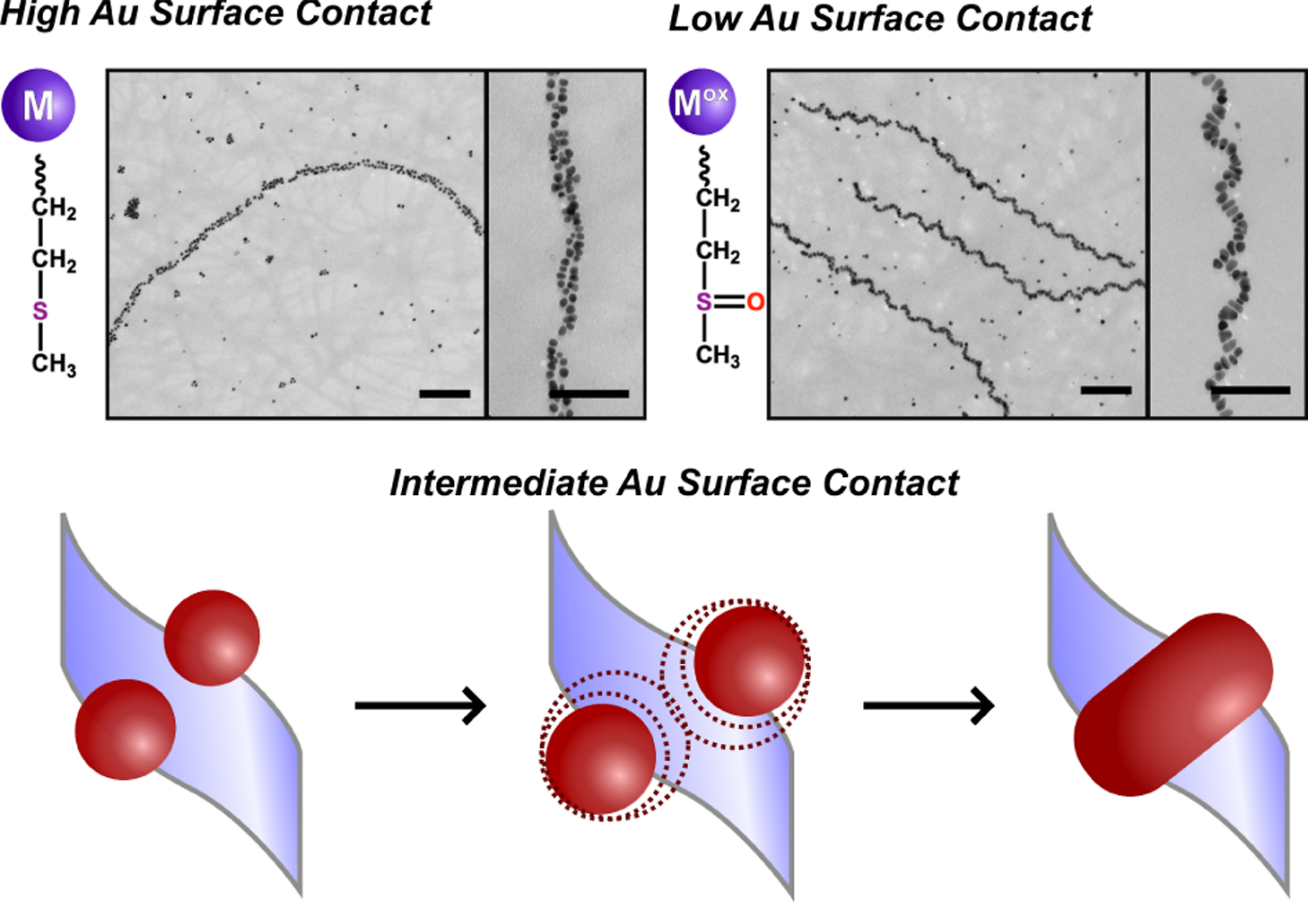
Structures formed from high-surface contact peptide sequences form double-helical superstructures (top left), while low-surface contact sequences yield Au NP single helices (top right). Scale bars 100 nm. Schematic illustration of proposed NP transformation with decreasing peptide–Au surface contact (bottom).

In a previous study, we determined that methionine oxidation leads to a decrease in the peptide–Au surface contact in the NP binding module.^17^ This decrease in surface contact correlates with a transition from double-helical assemblies of spherical Au NPs to single-helical assemblies of oblong Au NPs. Our results also showed that PEP_Au_ yielded spherical Au NPs, whereas PEP_Au_^M-ox^ yielded larger, non-spherical Au NPs, suggesting that a decrease in surface contact compromises the binding ability of the peptide capping ligand and leads to the formation of the oblong Au NPs.^17^ However, that study did not yield any insight into the origin of the transition from double- to single-helical assemblies. Notably, the fibers formed from C_16_-(PEP_Au_)_2_ and C_16_-(PEP_Au_^M-ox^)_2_ appear similar when imaged with TEM, and atomic force microscopy (AFM) images suggest both form helical ribbons (Figure S3). While C_16_-(PEP_Au_^M-ox^)_2_ fibers appear more tightly coiled than C_16_-(PEP_Au_)_2_ fibers (Figure S3c,d), the observed difference in NP assembly structure cannot solely be correlated with the observed differences in the fiber morphology, especially because the NP size and shape also change. Based on these observations, we hypothesize that the transition from double to single helices correlates with a decrease in the NP binding module’s Au surface contact. Specifically, we postulate that the double-helical superstructures may derive from the binding of two spherical NPs to the face of a helical ribbon fiber template (Figure 2). If the NP binding ability of the peptide decreases, particle growth would be less limited, resulting in the formation of larger oblong NPs across the face of the helical ribbon. Consequently, the NP superstructure would now be single helical.

**Figure 3:**
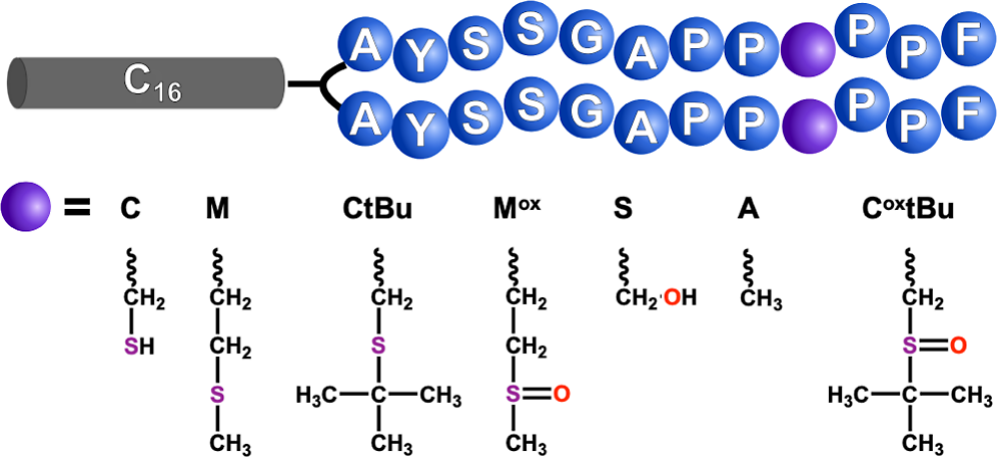
Family of amino acid modifications at the peptide ninth position, organized left to right from highest to lowest Au affinity.

To examine this possibility, we present here a family of divalent peptide conjugates in which we modify the sequence of the NP binding module to control its degree of contact with the Au surface. Studies have identified Y_2_, M_9_, and F_12_ as the primary anchoring residues in PEP_Au_, which allow it to serve as a NP non-covalent capping ligand.^29^ Based on our fiber assembly model, Y_2_ engages in β-sheet formation near the core of the assembled fibers and likely does not play a major role in binding NPs.^14,15,17^ M_9_ and F_12_ are in the particle binding module and play an integral role in anchoring NPs to the fibers. Because M_9_ oxidation results in a transition from double to single helices and decreases the NP surface contact of the NP binding module, we synthesized a series of peptide conjugates with different amino acids at the ninth position: C_16_-(AYSSGAPPXPPF)_2_, where X = cysteine C, methionine M, tertbutyl cysteine CtBu, alanine A, serine S, methionine sulfoxide M^ox^, and tertbutyl cysteine sulfoxide C^ox^tBu (Figures 3, S1, and S2). Sulfur-containing ligands, especially thiol functional groups, have strong associations with Au NPs on the level of covalent bonds;^32−34^ for the residues containing a sulfur atom, we gradually increased the steric bulk of the adjacent groups to inhibit binding. A and S were included because they have comparatively moderate–weak contact with Au surfaces.^35^ We reasoned that the NP binding affinity would decrease thusly: C > M > CtBu > M^ox^ ≅ A ≅ S > C^ox^tBu.

Based on our assembly model for this class of peptide conjugates, we predicted that all variants would readily form fibers in aqueous buffer, which was confirmed using TEM imaging (Figure S4). Fourier transform infrared (FTIR) and circular dichroism (CD) spectroscopy revealed some similarities in the molecular structure of the conjugates within this series of fibers. Each fiber sample displayed an amide I band of similar intensity at ∼1630 cm^–1^ in the FTIR spectrum, which is indicative of the β-sheet secondary structure.^9,36^ In addition, a sharp symmetric (CH_2_) band at ∼2850 cm^–1^ was observed for all samples, indicating ordered packing of the aliphatic tails (Figure S5).^9,37^ The CD spectra across the series were less homogeneous, and each spectrum likely reflects contributions from more than one type of secondary structure, as we have reported in previous studies of analogous conjugates.^15,16^ The S and A variants display a strong and broad negative feature from ∼210–220 nm, which is consistent with the β-sheet secondary structure (Figure S6a).^38,39^ In the case of the A variant, a shift in this feature to lower wavelength could be attributed to strong contributions from the PPII secondary structure.^40,41^ The C, M, and M^ox^ variants display a negative feature at ∼205 nm, which can be assigned to the PPII secondary structure, and the broadening of this primary peak is likely due to contributions from the β-sheet secondary structure (Figure S6b), which is supported by the FTIR data discussed above. We cannot definitively interpret the CD spectra for the C(*t*Bu) and C^ox^(*t*Bu) variants (Figure S6c), yet from the FTIR data, we know that these variants form fibers that have some β-sheet characteristic. The significant steric bulk introduced by the *t*Bu group could significantly disrupt secondary structure formation at the C-terminus, resulting in more ambiguous CD spectra. In summary, we can conclude that all variants exhibit a β-sheet secondary structure, while varying the ninth position can affect the C-terminal structure.

**Figure 4:**
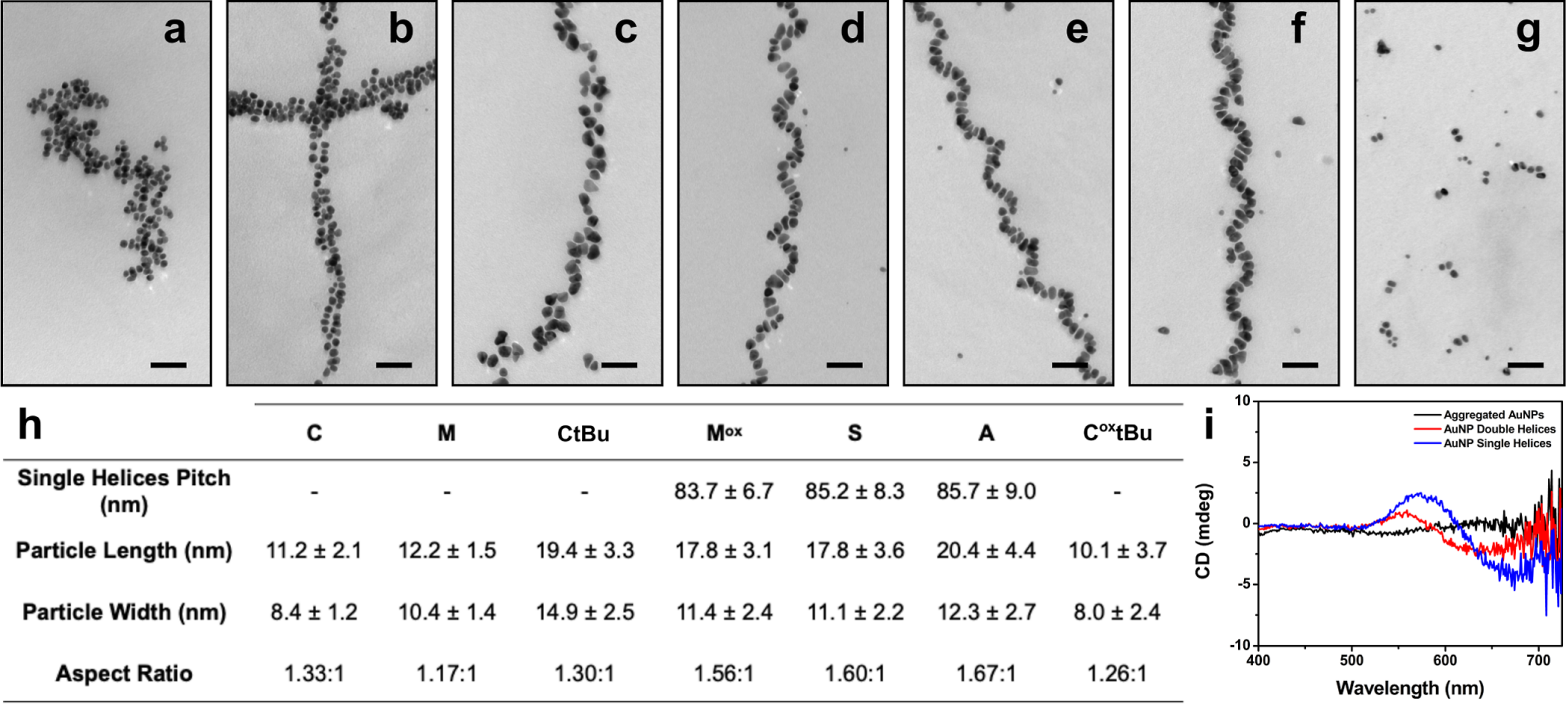
(a) Au NP aggregates produced using C_16_-(AYSSGAPPCPPF)_2_; (b) double-helical superstructures produced using C_16_-(AYSSGAPPMPPF)_2_; (c) C_16_-(AYSSGAPPCtBuPPF)_2_ yields a blend of single- and double-helical assemblies; single helices produced using (d) C_16_-(AYSSGAPPM^ox^PPF)_2_, (e) C_16_-(AYSSGAPPSPPF)_2_, and (f) C_16_-(AYSSGAPPAPPF)_2_; and (g) discrete Au NPs formed using C_16_-(AYSSGAPPC^ox^tBuPPF)_2_. Scale bars 50 nm. (h) Table of NP dimensions and helix pitch (where applicable). (i) CD spectra for the three categories of superstructures: aggregated NPs, double helices, and single helices.

Prior to NP assembly experiments, we first verified that discrete Au NPs could be formed using each of the amine-terminated peptide variants as the capping ligand (Figures S7 and S8 and Table S1). We next subjected the family of conjugates to our established Au NP synthesis and single helix assembly conditions.^15−18^ The C variant was predicted to have the strongest contact with the Au NPs, and it yielded aggregates of spherical NPs bearing some apparent underlying structure which is too irregular to assign (Figure 4a, S9). M was expected to have a lower NP affinity than C, and this variant produced linear superstructures of spherical NPs with some double-helical characteristic, as we reported previously (Figures 4b and S10).^14,15^ Protecting the cysteine thiol with a tertbutyl group should decrease its Au surface contact significantly, and the NP superstructures formed using the C*t*Bu variant are best described as a blend of double and single helices (Figure 4c and S11). The M^ox^, A, and S variants are considered “moderate” Au binders, and each yielded NP single helices composed of oblong NPs (Figures 4d–f and S12–S14). Last, oxidizing the tertbutyl-protected cysteine residue introduces significant steric bulk at the C-terminus, and while this variant does form fibers in aqueous assembly buffer, the extra bulk apparently inhibits attachment of NP to the fibers (Figures 4g and S15). Across this series from “strong binding” to “weak binding”, the aspect ratio of the assembled NP generally increased (Figure 4h), and the superstructures transitioned from NP aggregates to double helices and then to single helices; in the case of the C^ox^tBu variant, the NPs were spherical yet not assembled onto fibers. Accompanying this transition in structure is the appearance of a plasmonic chiroptical signal going from the aggregates to the single-helical assemblies, illustrating how adjustments to molecular structure can lead to emergence of unique collective plasmonic properties (Figure 4i). Such properties are relevant to a variety of applications from sensing to optics.^12,42−46^

REST-MD simulations were used to explore our proposed connection between Au-peptide binding strength and the ability to support either single- or double-helix assembles. These simulations predict the likely conformational ensemble of each peptide in the surface-adsorbed state at the aqueous Au interface (examples shown in Figures 4c,d and S16–S22). Based on these simulation data, the degree of binding between the residues of the PEP_Au_ peptide and its six variants with the aqueous Au interface was evaluated, with particular emphasis on the residues in the C-terminal “particle binding” module. To do this, we computed a binding score for each residue in each peptide, where the score was defined as the fraction of the trajectory for which each residue was deemed in contact with the Au surface (denoted the contact fraction, expressed as number between 0 and 1) and the Au binding free energy of the counterpart amino acid for that particular residue. Most of these free energy amino acid data have been published previously but were not available for the amino acid analogues M^ox^, C*t*Bu, and C^ox^*t*Bu. These new data were generated as part of the current work using umbrella sampling simulations; the full set of contact fraction data and amino acid binding free energy data, along with the resultant binding scores, are provided in the Supporting Information (Tables S3–S6).

**Figure 5:**
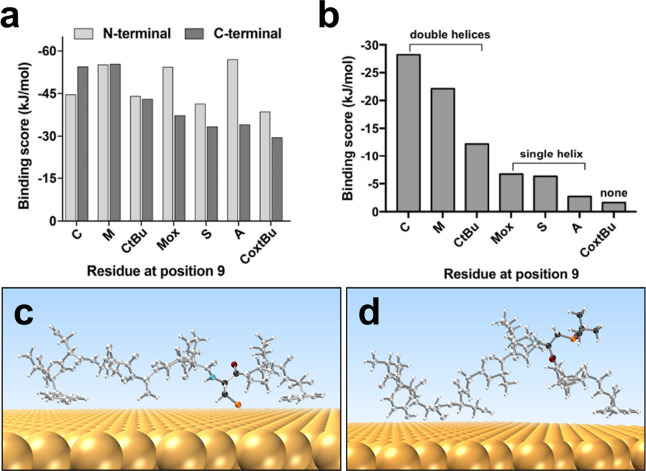
Binding scores for the original PEP_Au_ sequence (residue M at position 9) and the six sequence variants. (a) Sum of residue–surface binding scores for the N-terminal half (residues 1–6) and the C-terminal half (residues 7–12). (b) Residue–surface binding score for the residue at the ninth position in the sequence. Representative structures of (c) 9C and (d) 9C^ox^tBu peptides adsorbed on the Au(111) surface according to REST-MD simulations, corresponding to highest and lowest binding scores, respectively. C and C^ox^tBu are highlighted with color: C, dark gray; H, light gray; N, blue; O, dark red; and S, orange.

The binding scores for each residue can be summed over a given range of the peptide sequence to determine a cumulative binding score. As anticipated, the binding score summed over the N-terminal half of the sequence (Figure 5a) did not show any correlation with the propensity to form single-, double-, or no-helix assemblies. However, the sum over the C-terminal half (residues 7–12, the particle binding module) revealed a trend in binding score (Figure 5a) that was approximately consistent with the experimentally observed propensity to form double-, single-, or no-helix-based assemblies. Following the hypothesis proposed in earlier work regarding the contribution of the residue at position 9 in the sequence,^17,35^ the binding score exclusively for the residue at position 9 (Figure 5b) was considered, revealing a strong correlation with the structural traits of the associated assembly.

A possible explanation for this clear trend in surface binding strength at position 9 of the sequence as a function of variant can be attributed to the conformational recalcitrance of the C-terminal region of the peptide with respect to variation of the residue at position 9. In other words, each of the variants was found to maintain at least some conformational similarity with respect to the original sequence. To quantify this, the conformational ensemble of each variant adsorbed at the aqueous Au interface was characterized using a clustering analysis. In brief, in this analysis, conformations that are sampled by the REST-MD simulation are grouped together (into clusters) on the basis of similarity in the peptide backbone structure. These simulation data can also be used to determine the most common secondary structure(s) of each peptide in relation to the Au(111) surface. The structures of 9C and 9C^ox^tBu (highest and lowest scores, respectively) are shown in Figure 5c,d, respectively. The ninth position amino acid is colored for clarity, showing how the cysteine residue of 9C closely associates with the Au(111) surface, while C^ox^tBu is directed away from the surface with no apparent contact (images for the remaining peptide sequences except 9A can be found in Figures S16–S22). The clustering analysis yields the number of clusters and the population of each cluster. Typically, the top five most populated clusters capture the majority of the ensemble. The cluster centroid is the structure that best represents each cluster conformation; on that basis, the cluster centroids were compared for the top five clusters between the original PEP_Au_ peptide and the six variants. This comparison (data in Figure S26) revealed the structural similarity of each variant with PEP_Au_ in the surface-adsorbed state. These data suggest that the success of the substitution strategy at position 9 is due in part to the fact that variation in the ninth residue does not result in a substantial departure from the surface-bound conformational ensemble of PEP_Au_.

**Figure 6:**
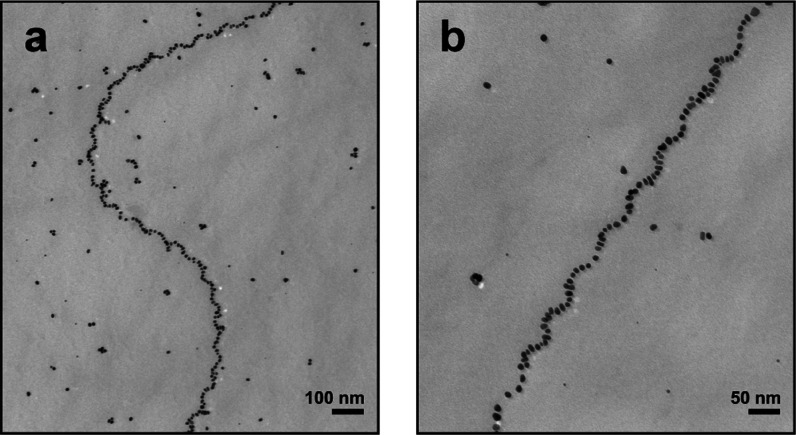
Single helices produced using C_16_-(AYSSGAPPTPPF)_2_.

Previously published data regarding the binding free energies of amino acids at the aqueous Au interface^35^ suggest a range of residues for substitution at position 9 that might be able to support a residue binding score in the single-helix range (−1 to −10 kJ mol^–1^): proline, threonine, leucine, aspartic acid, glutamic acid, and lysine. A substitution of methionine with a charged residue (i.e., aspartic acid, glutamic acid, or lysine) may produce a strong conformational change of the peptide, thereby potentially disrupting the conformational recalcitrance proposed above; proline was excluded due to the abundance of proline already present in the C-terminal half of the sequence. We elected to test the threonine variant (9T) because it is structurally similar to serine. This variant was computationally modeled using REST-MD simulations. The binding score analysis determined a binding score of −4.5 kJ mol^–1^ for 9T, which falls between the 9A and 9S (Figure 4b). Based on this evidence, we prepared the 9T variant (Figures S23 and S24) and conducted Au NP synthesis and assembly experiments. In line with our prediction, it yielded well-defined single helices (Figures 6 and S25).

## Conclusions

Variation of the ninth amino acid within the AuNP binding module of C_16_-(PEP_Au_)_2_ yielded a family of peptide conjugates with differential AuNP binding affinities which were used to prepare a series of NP assemblies that represent snapshots of the transition from double- to single-helical Au NP superstructures. Our experimental observations coupled with simulations that predict a “binding score” for each peptide variant provide compelling evidence that the relative Au NP binding affinity of the peptides significantly influences the helical morphology of the superstructure and governs the double- to single-helical structural transformation. Accompanying this structural transition is the emergence of observable plasmonic chiroptical behavior for the single helices. These results and insights demonstrate that single amino acid modifications to the NP binding module of the PEP_Au_ sequence can result in dramatic changes to the structure and properties of helical NP assemblies. A significant implication of these results is that small adjustments in molecular chemistry can be advantageously used to precisely control the nano- and microscale structure and collective properties of NP superstructures.

## Supporting Information Available

LC–MS, CD, FTIR, AFM, additional TEM, and simulation data (PDF)
