# Supplementary material for: Single Amino Acid Modifications for Controlling the Helicity of Peptide-Based Chiral Gold Nanoparticle Superstructures
Source: J Am Chem Soc. 2023 Mar 13;145(11):6546–53. doi: 10.1021/jacs.3c00827 (PMC10037318; doi:10.1021/jacs.3c00827)
Supplement: Supplementary file 1 — ja3c00827_si_001.pdf [file ja3c00827_si_001.pdf]

Supporting Information

Single Amino Acid Modifications for Controlling the Helicity of Peptide-Based Chiral Gold Nanoparticle Superstructures

Sydney C. Brooks,[†] Ruitao Jin,[◊,§] Victoria C. Zerbach,[†] Yuyu Zhang,[†] Tiffany R. Walsh,*[◊] and Nathaniel L. Rosi*[†‡]

[†] Department of Chemistry, University of Pittsburgh, Pittsburgh, Pennsylvania 15260, United States
[‡] Department of Chemical and Petroleum Engineering, University of Pittsburgh, Pennsylvania 15260, United States
[◊] Institute for Frontier Materials, Deakin University, Geelong, VIC 3216, Australia
[§] Current Address: Research School of Biology, Australian National University, Canberra, ACT, 2600, Australia

Table of Contents

1. Peptide Conjugate Synthesis and Characterization....2
2. Peptide Conjugate Assembly Studies....4
3. Synthesis of Amine-Terminated Peptide Variants and NP Synthesis Studies....6
4. Nanoparticle Assembly Studies....8
5. Representative Structures of Peptide Variants on Au(111) Surface....12
6. 9T Peptide Conjugate Synthesis and NP Assembly Studies....16
7. Computational Methodology and Supporting Data....18
8. References....27

1. Peptide Conjugate Synthesis and Characterization

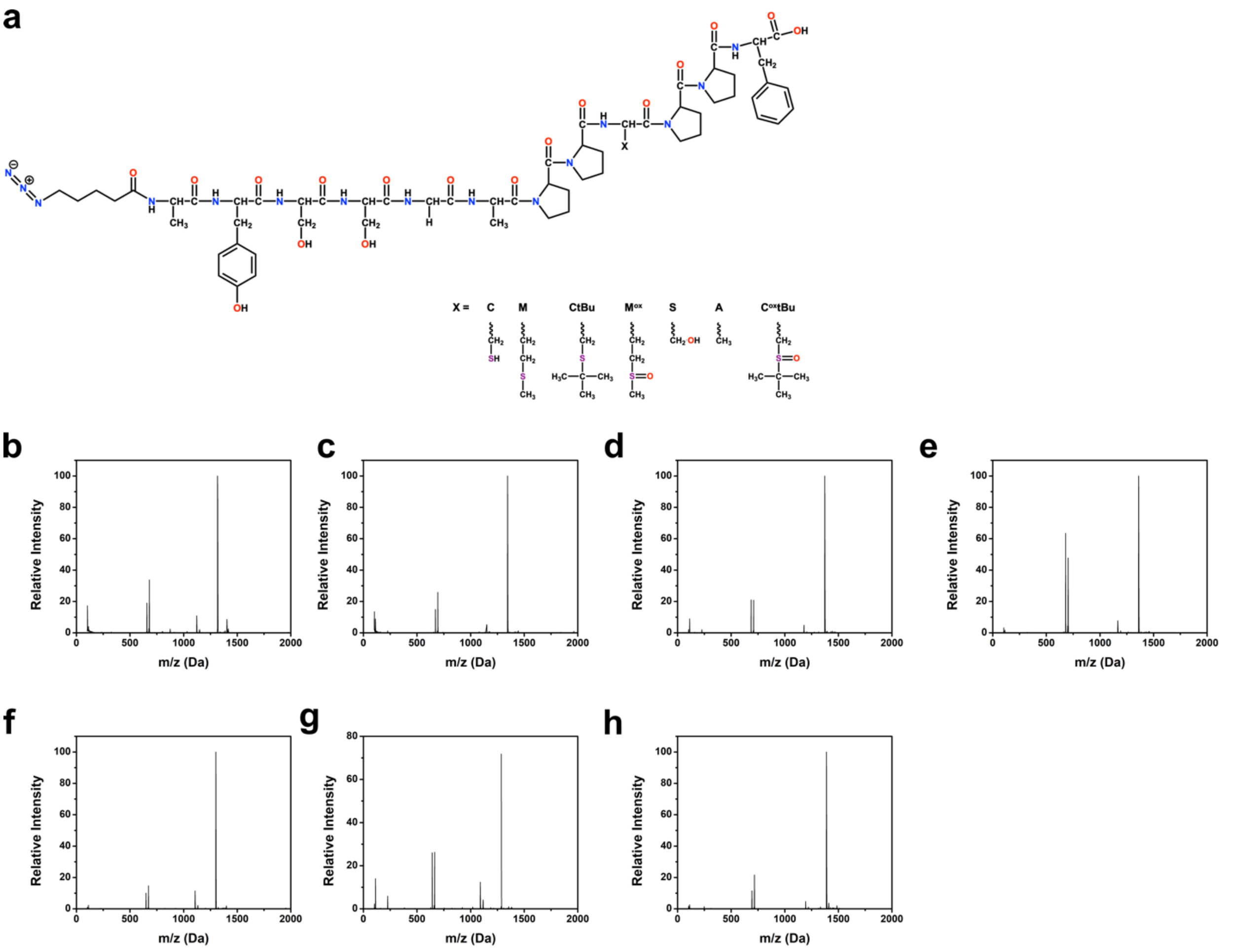

Figure S1. (a) Representative molecular structure of N_3-AYSSGAPPXPPF. LC-MS mass assignment of (b) N_3-AYSSGAPPCPPF, m/z = 1318 Da (M-H$^+$); 682 Da (M-2H$^+$+ HCOO$^-$)/2; 658 Da (M-2H$^+$)/2; (c) N_3-AYSSGAPPMPPF, m/z = 1347 Da (M-H$^+$); 696 Da (M-2H$^+$+ HCOO$^-$)/2; 672 Da (M-2H$^+$)/2; (d) N_3-AYSSGAPPCtBuPPF, m/z = 1374 Da (M-H$^+$); 710 Da (M-2H$^+$+ HCOO$^-$)/2; 686 Da (M-2H$^+$)/2; (e) N_3-AYSSGAPPM^{ox}PPF, m/z = 1363 Da (M-H$^+$); 704 Da (M-2H$^+$+ HCOO$^-$)/2; 680 Da (M-2H$^+$)/2; (f) N_3-AYSSGAPPSPPF, m/z = 1302 Da (M-H$^+$); 674 Da (M-2H$^+$+ HCOO$^-$)/2; 650 Da (M-2H$^+$)/2; (g) N_3-AYSSGAPPAPPF, m/z = 1286 Da (M-H$^+$); 666 Da (M-2H$^+$+ HCOO$^-$)/2; 642 Da (M-2H$^+$)/2; (h) N_3-AYSSGAPPC^{ox}tBuPPF, m/z = 1390 Da (M-H$^+$); 718 Da (M-2H$^+$+ HCOO$^-$)/2; 694 Da (M-2H$^+$)/2.

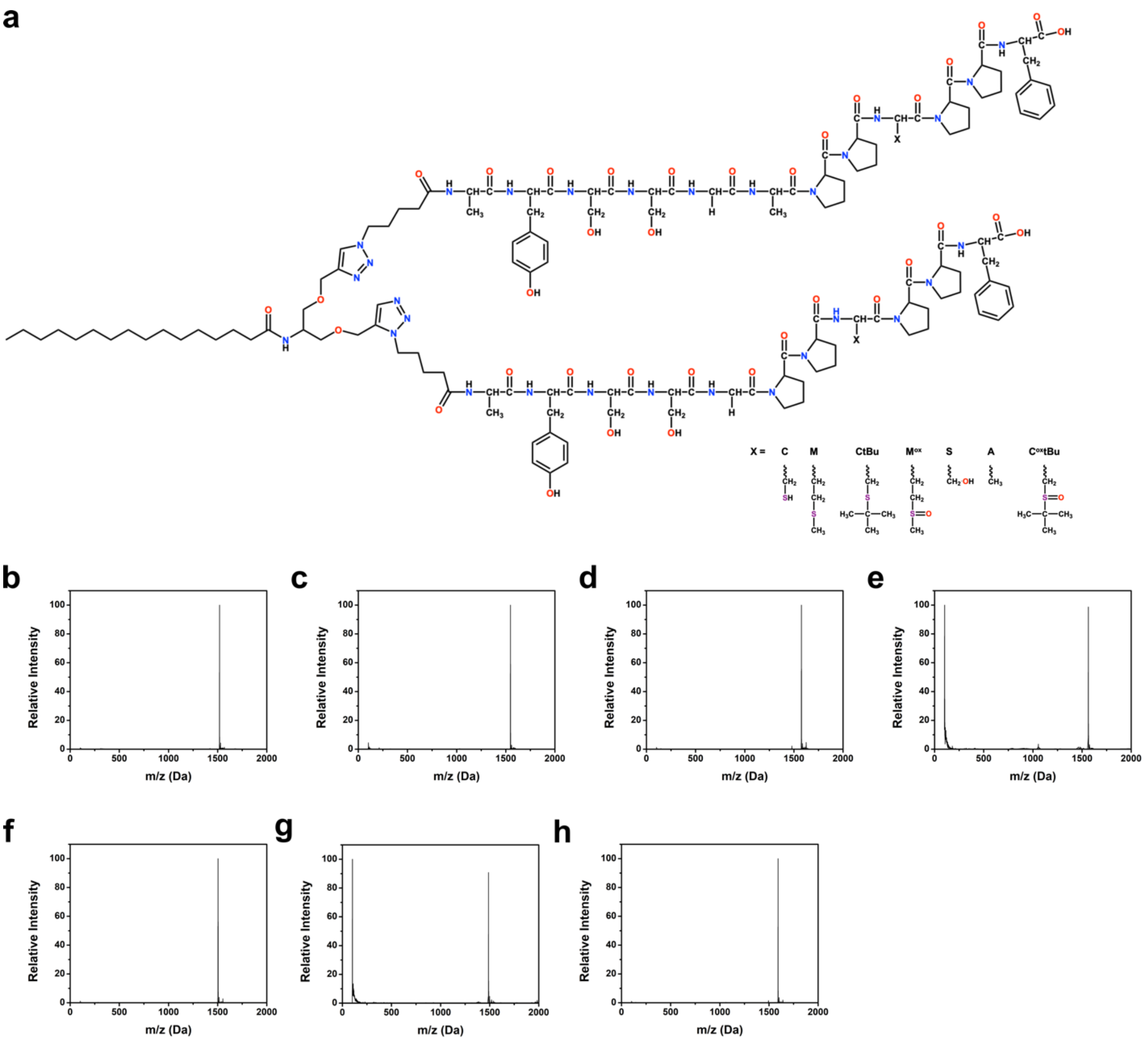

Figure S2. (a) Representative molecular structure of C$_{16}$-(AYSSGAPPXPPF)$_2$. LC-MS mass assignment of (b) C$_{16}$-(AYSSGAPPCPPF)$_2$, m/z 1520 Da (M-2H$^+$)/2; (c) C$_{16}$-(AYSSGAPPMPPF)$_2$, m/z 1548 Da (M-2H$^+$)/2; (d) C$_{16}$-(AYSSGAPPCtBuPPF)$_2$, m/z 1576 Da (M-2H$^+$)/2; (e) C$_{16}$-(AYSSGAPPM^{ox}PPF)$_2$, m/z 1565 Da (M-2H$^+$)/2; (f) C$_{16}$-(AYSSGAPPSPPF)$_2$, m/z 1504 Da (M-2H$^+$)/2; (g) C$_{16}$-(AYSSGAPPAPPF)$_2$, m/z 1488 Da (M-2H$^+$)/2; (h) C$_{16}$-(AYSSGAPPC^{ox}tBuPPF)$_2$, m/z 1592 Da (M-2H$^+$)/2.

2. Peptide Conjugate Assembly Studies

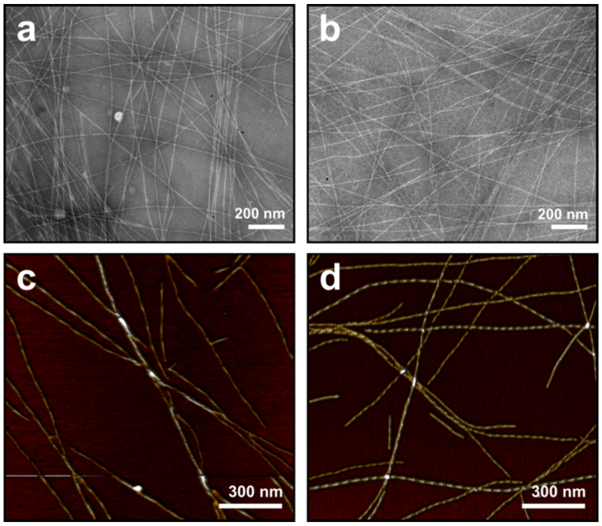

Figure S3. TEM and AFM images of C_{16}-(AYSSGAPPMPPF)$_2$ (a,c) and C_{16}-(AYSSGAPPM^{ox}PPF)$_2$ (b,d).

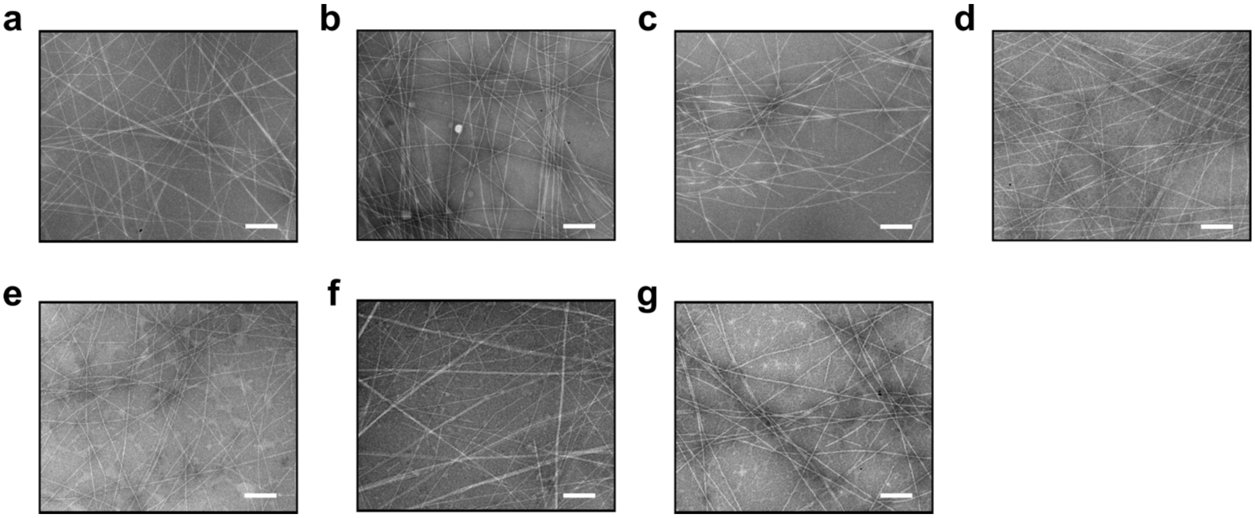

Figure S4. TEM images of peptide conjugate fibers assembled from (a) C_{16}-(AYSSGAPPCPPF)$_2$, (b) C_{16}-(AYSSGAPPMPPF)$_2$, (c) C_{16}-(AYSSGAPPCtBuPPF)$_2$, (d) C_{16}-(AYSSGAPPM^{ox}PPF)$_2$, (e) C_{16}-(AYSSGAPPSPPF)$_2$, (f) C_{16}-(AYSSGAPPAPPF)$_2$, and (g) C_{16}-(AYSSGAPPC^{ox}tBuPPF)$_2$. Scale bars 200 nm.

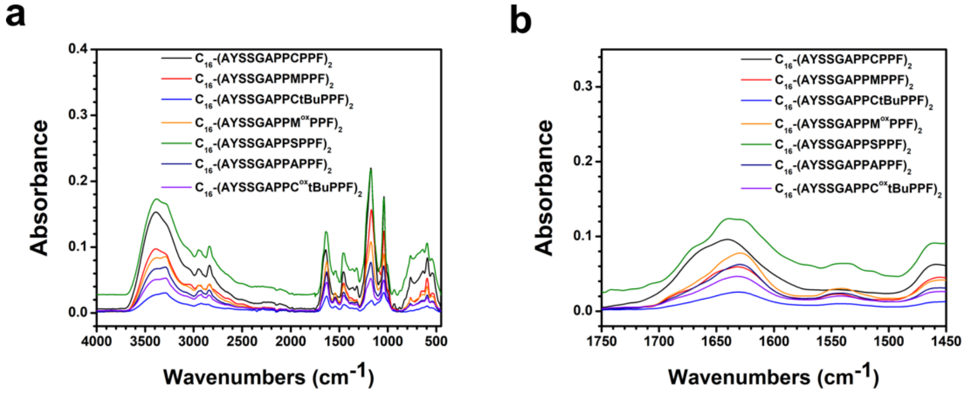

Figure S5. ATR-FTIR spectra of peptide conjugate fibers showing (a) the full wavelength range and (b) expanded view of the amine region.

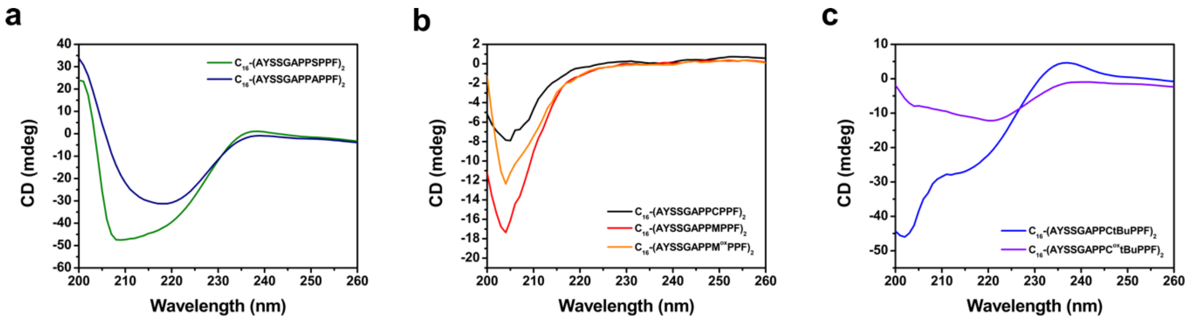

Figure S6. CD spectra of (a) C_{16}-(AYSSGAPPSPPF)$_2$ and C_{16}-(AYSSGAPPSPPF)$_2$; (b) C_{16}-(AYSSGAPPCPPF)$_2$, C_{16}-(AYSSGAPPMPPF)$_2$, and C_{16}-(AYSSGAPPM^{ox}PPF)$_2$; and (c) C_{16}-(AYSSGAPPCtBuPPF)$_2$ and C_{16}-(AYSSGAPPC^{ox}tBuPPF)$_2$.

3. Synthesis of Amine-Terminated Peptide Variants and NP Synthesis Studies

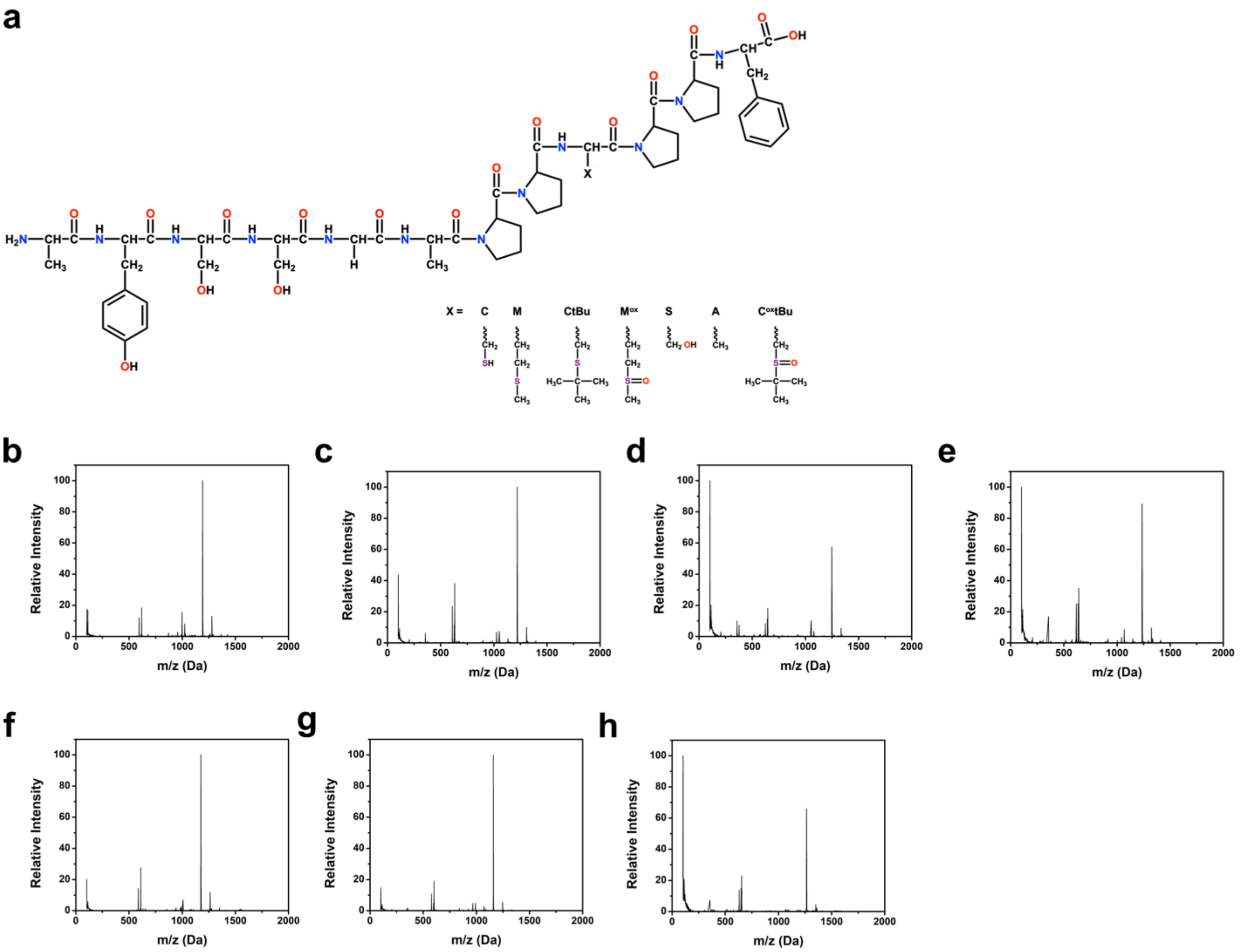

Figure S7. (a) Representative molecular structure of NH_2-AYSSGAPPXPPF. LC-MS mass assignment of (b) NH_2-AYSSGAPPCPPF, m/z = 1193 Da ($M-H^+$); 619 Da ($M-2H^+ + HCOO^-$)/2; 596 Da ($M-2H^+$)/2; (c) NH_2-AYSSGAPPMPPF, m/z = 1221 Da ($M-H^+$); 633 Da ($M-2H^+ + HCOO^-$)/2; 610 Da ($M-2H^+$)/2; (d) NH_2-AYSSGAPPCtBuPPF, m/z = 1249 Da ($M-H^+$); 647 Da ($M-2H^+ + HCOO^-$)/2; 623 Da ($M-2H^+$)/2; (e) NH_2-AYSSGAPPM^{ox}PPF, m/z = 1237 Da ($M-H^+$); 641 Da ($M-2H^+ + HCOO^-$)/2; 618 Da ($M-2H^+$)/2; (f) NH_2-AYSSGAPPSPPF, m/z = 1177 Da ($M-H^+$); 611 Da ($M-2H^+ + HCOO^-$)/2; 588 Da ($M-2H^+$)/2; (g) NH_2-AYSSGAPPAPPF, m/z = 1161 Da ($M-H^+$); 603 Da ($M-2H^+ + HCOO^-$)/2; 580 Da ($M-2H^+$)/2; (h) NH_2-AYSSGAPPC^{ox}tBuPPF, m/z = 1265 Da ($M-H^+$); 655 Da ($M-2H^+ + HCOO^-$)/2; 632 Da ($M-2H^+$)/2.

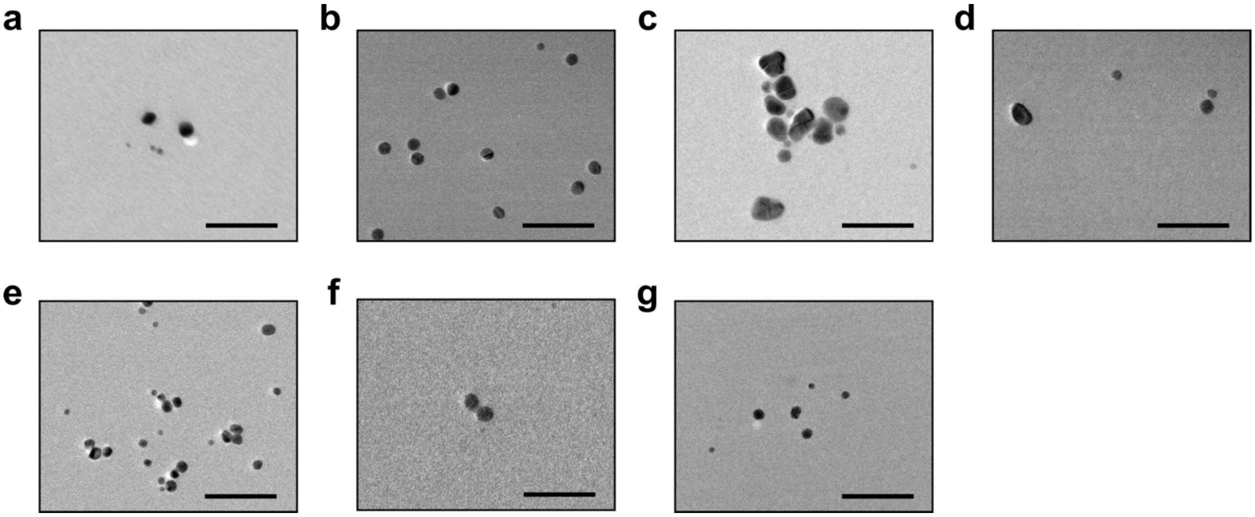

Figure S8. AuNPs produced from of NH_2-AYSSGAPPXPPF, where X = (a) C, (b) M, (c) CtBu, (d) M^{ox}, (e) S, (f) A, and (g) C^{ox}tBu. Scale bars are 50 nm.

Table S1. Particle length and width of AuNPs synthesized by amine-terminated peptide variants.

	**C**	**M**	**CtBu**	**M^{ox}**	**S**	**A**	**C^{ox}tBu**
**Particle Length (nm)**	6.6 ± 3.7	9.6 ± 1.4	14.2 ± 7.4	7.6 ± 3.0	7.3 ± 2.0	4.6 ± 1.8	7.4 ± 2.1
**Particle Width (nm)**	6.4 ± 3.3	8.7 ± 1.2	12.4 ± 5.7	6.6 ± 1.9	6.3 ± 1.6	4.1 ± 1.4	6.7 ± 1.7

4. Nanoparticle Assembly Studies

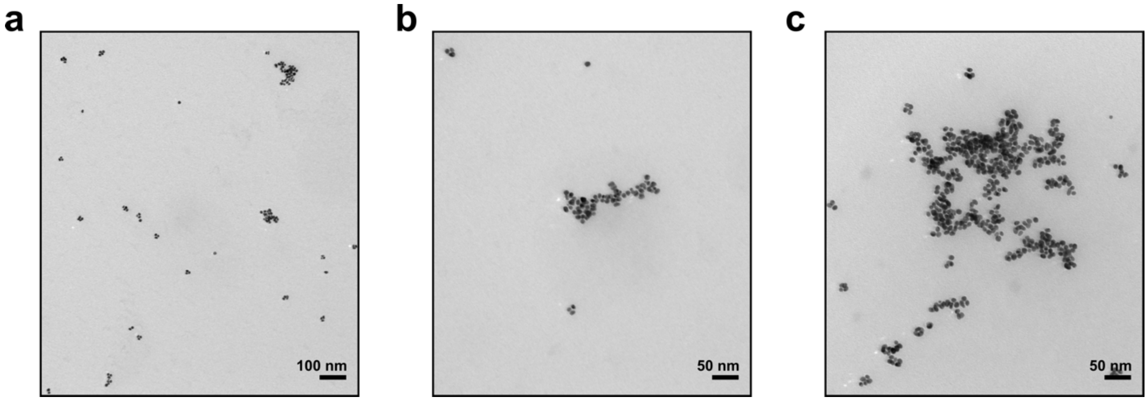

Figure S9. Additional TEM images of AuNP assemblies produced by C_{16}-(AYSSGAPPCPPF)$_2$.

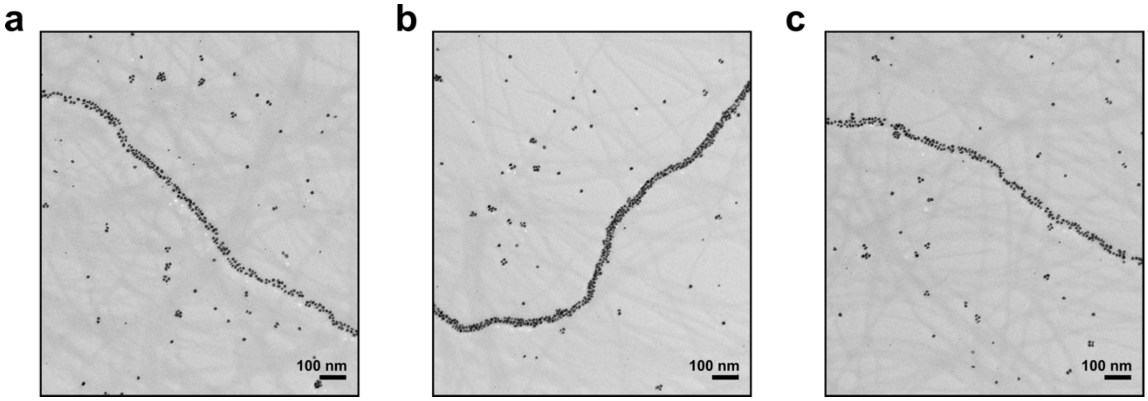

Figure S10. Additional TEM images of AuNP assemblies produced by C_{16}-(AYSSGAPPMPPF)$_2$.

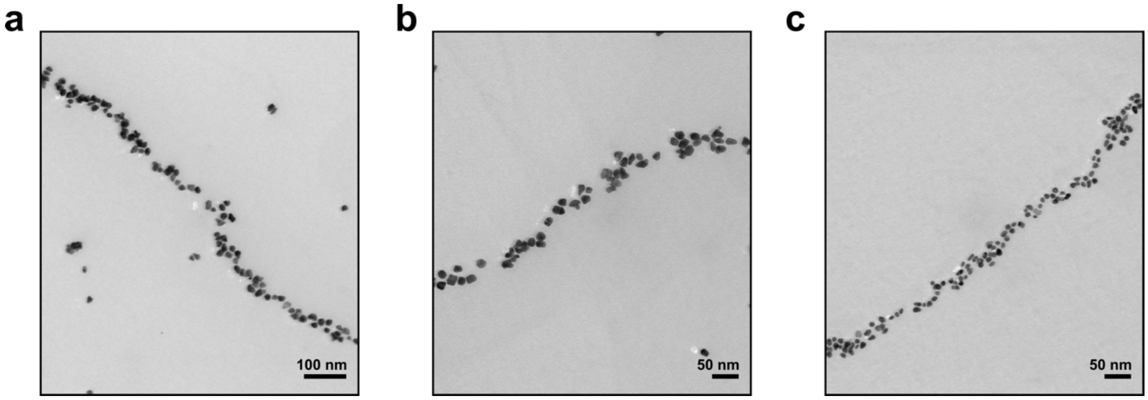

Figure S11. Additional TEM images of AuNP assemblies produced by C_{16}-(AYSSGAPPCtBuPPF)$_2$.

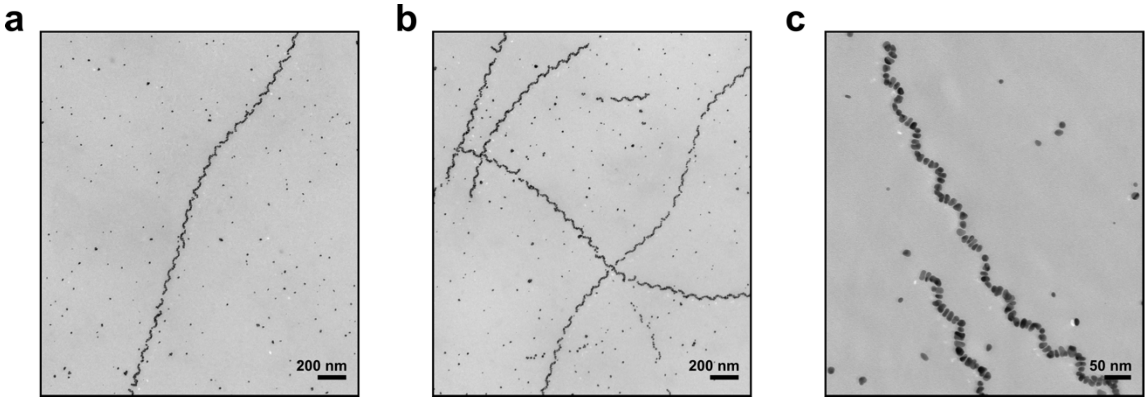

Figure S12. Additional TEM images of AuNP assemblies produced by C_{16}-(AYSSGAPPM^{ox}PPF)$_2$.

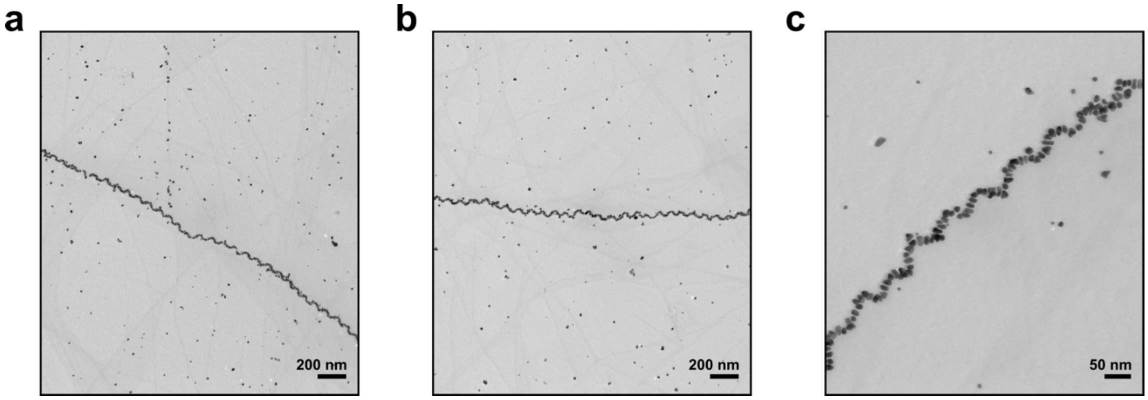

Figure S13. Additional TEM images of AuNP assemblies produced by C_{16}-$(AYSSGAPPSPPF)_2$.

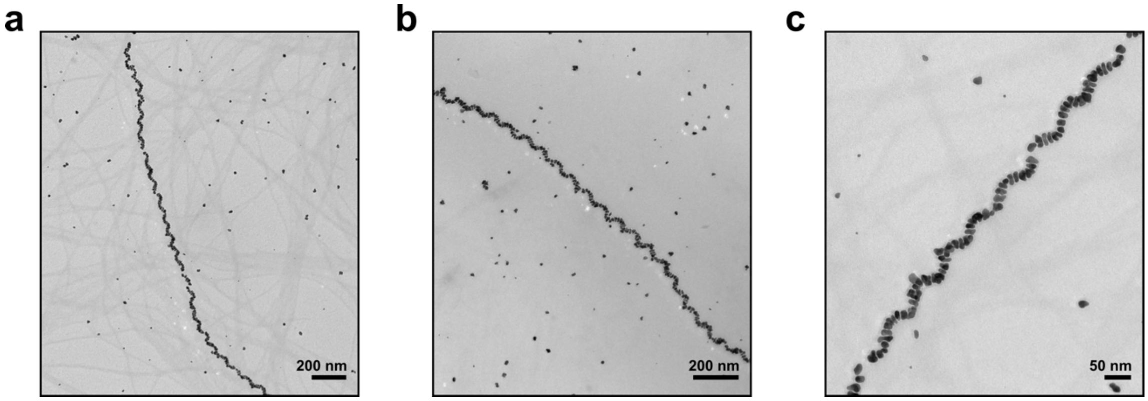

Figure S14. Additional TEM images of AuNP assemblies produced by C_{16}-$(AYSSGAPPAPPF)_2$.

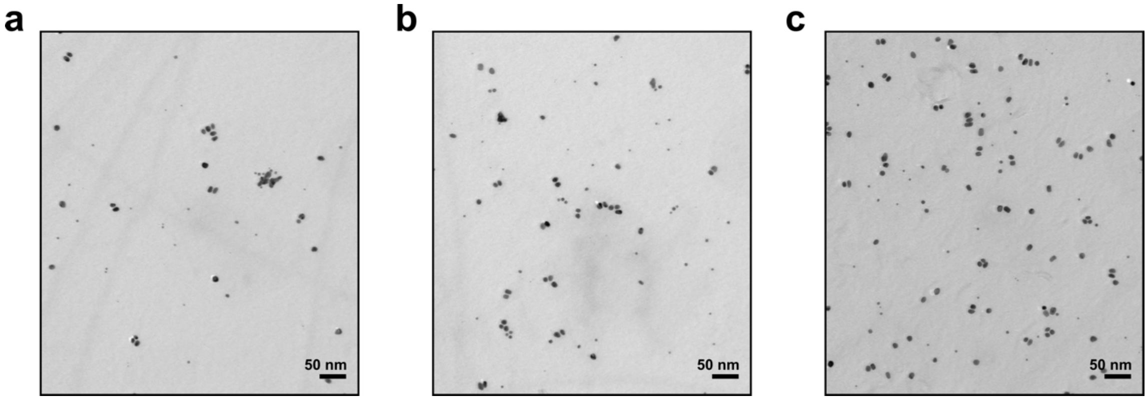

Figure S15. Additional TEM images of AuNP assemblies produced by C_{16}-(AYSSGAPPC^{ox}tBuPPF)$_2$.

5. Representative Structures of Peptide Variants on Au(111) Surface

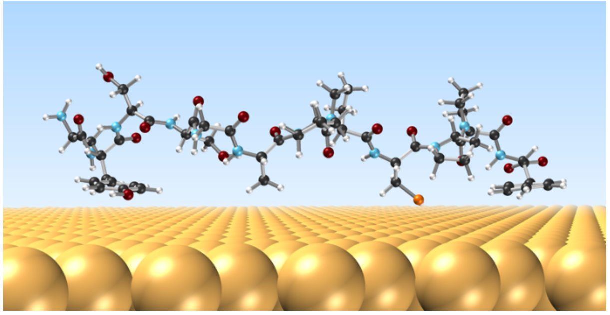

Figure S16. Representative structure of 9C peptide adsorbed on Au(111) surface according to REST-MD simulations (C, dark grey; H, light grey; N, blue; O, dark red; S, orange).

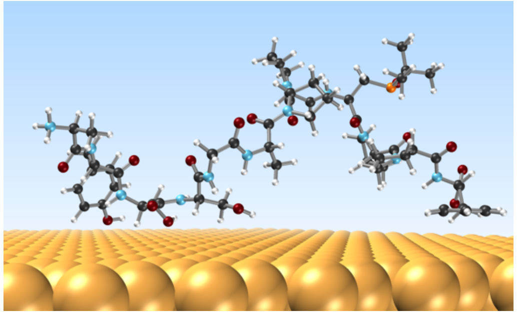

Figure S17. Representative structure of $9C^{ox}$tBu peptide adsorbed on Au(111) surface according to REST-MD simulations (C, dark grey; H, light grey; N, blue; O, dark red; S, orange).

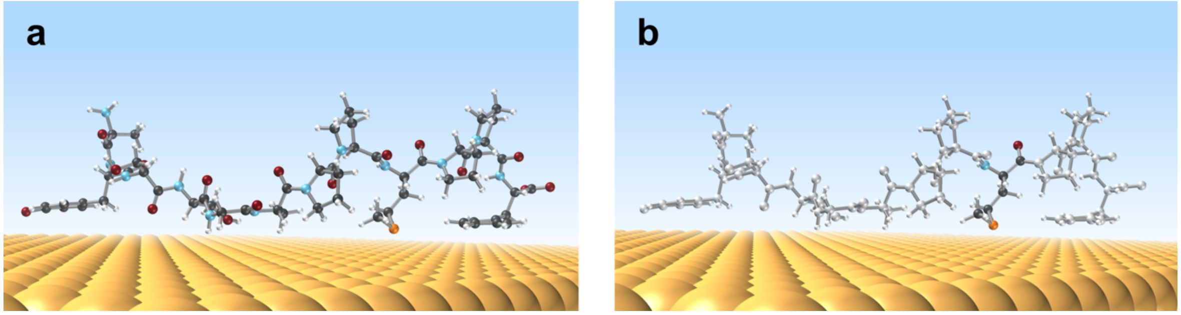

Figure S18. Representative structure of 9M peptide adsorbed on Au(111) surface according to REST-MD simulations (C, dark grey; H, light grey; N, blue; O, dark red; S, orange). In a), all atoms are colored, while in b) only the atoms of the 9th amino acid are colored for emphasis.

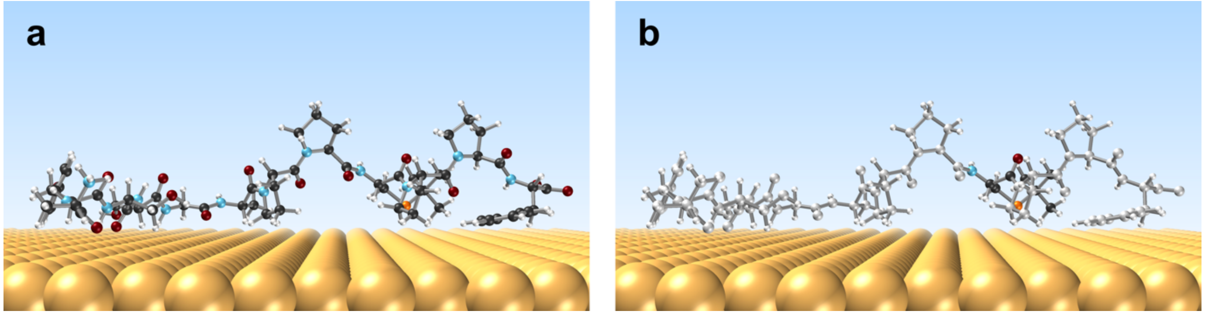

Figure S19. Representative structure of 9CtBu peptide adsorbed on Au(111) surface according to REST-MD simulations (C, dark grey; H, light grey; N, blue; O, dark red; S, orange). In a), all atoms are colored, while in b) only the atoms of the 9th amino acid are colored for emphasis.

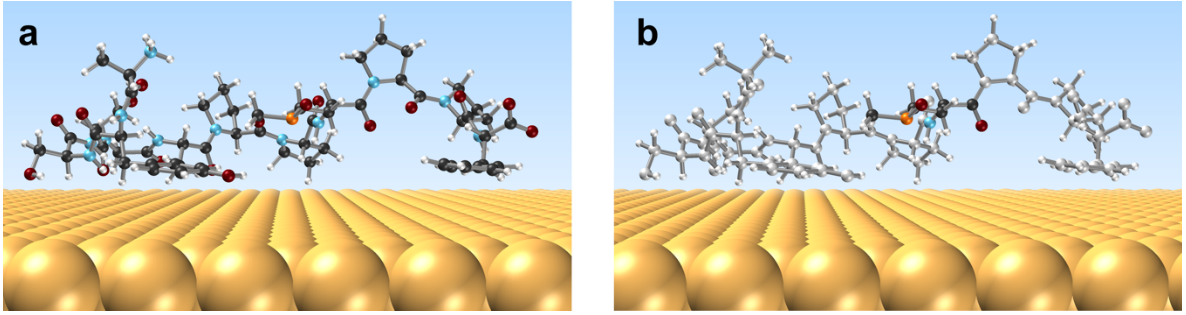

Figure S20. Representative structure of 9M^{ox} peptide adsorbed on Au(111) surface according to EST-MD simulations (C, dark grey; H, light grey; N, blue; O, dark red; S, orange). In a), all atoms are colored, while in b) only the atoms of the 9th amino acid are colored for emphasis.

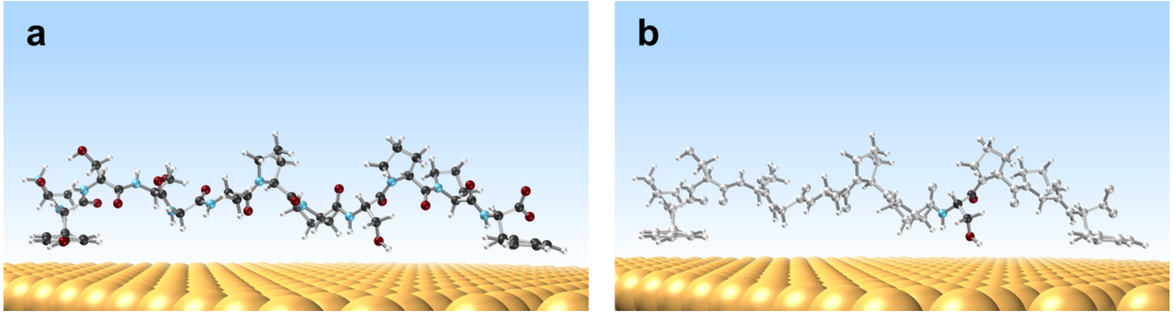

Figure S21. Representative structure of 9S peptide adsorbed on Au(111) surface according to REST-MD simulations(C, dark grey; H, light grey; N, blue; O, dark red). In a), all atoms are colored, while in b) only the atoms of the 9th amino acid are colored for emphasis.

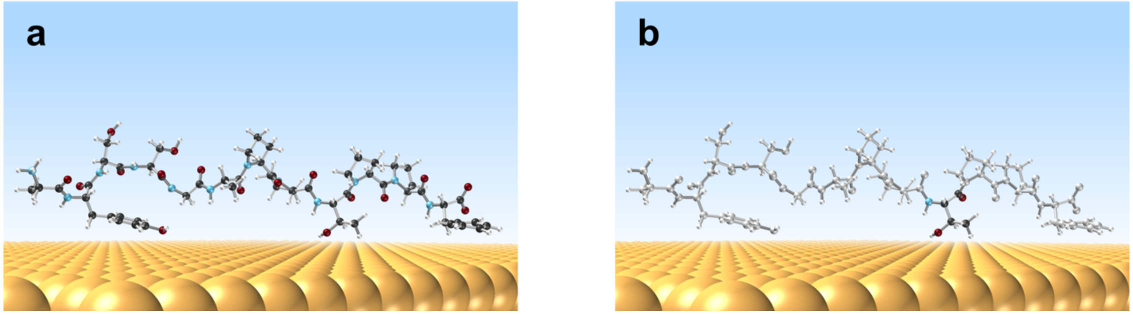

Figure S22. Representative structure of 9T peptide adsorbed on Au(111) surface according to REST-MD simulations (C, dark grey; H, light grey; N, blue; O, dark red). In a), all atoms are colored, while in b) only the atoms of the 9th amino acid are colored for emphasis.

6. 9T Peptide Conjugate Synthesis and NP Assembly Studies

a

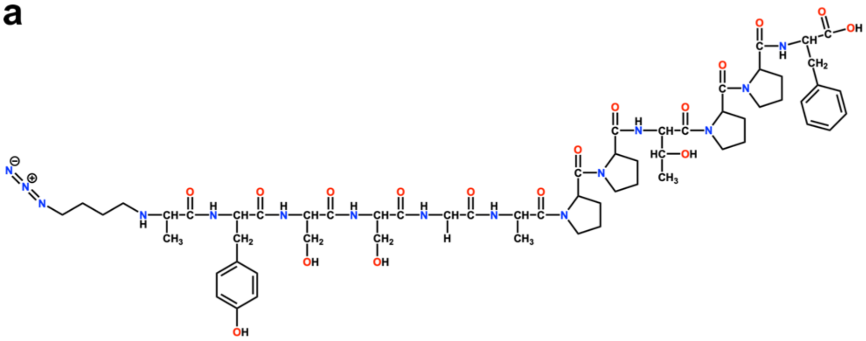

b

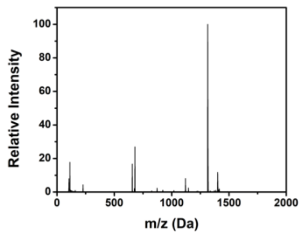

Figure S23. (a) Molecular structure and (b) LC-MS mass assignment of N_3-AYSSGAPPTPPF, m/z = 1316 Da (M-H$^+$); 681 Da (M-2H$^+$+ HCOO$^-$)/2; 657 Da (M-2H$^+$)/2.

a

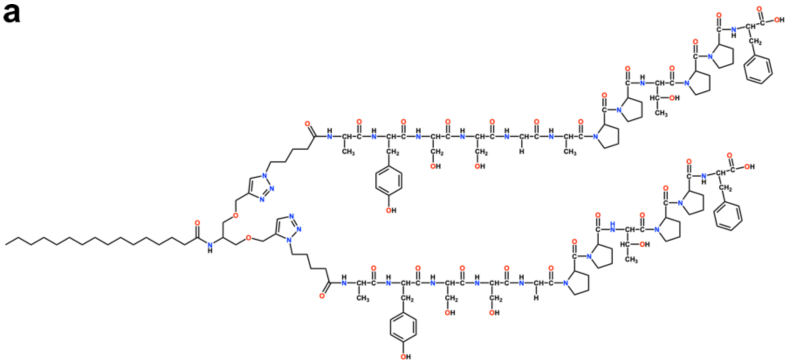

b

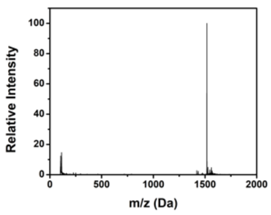

Figure 24. (a) Molecular structure and (b) LC-MS mass assignment of C_{16}-(AYSSGAPPTPPF)$_2$, m/z 1518 Da (M-2H$^+$)/2.

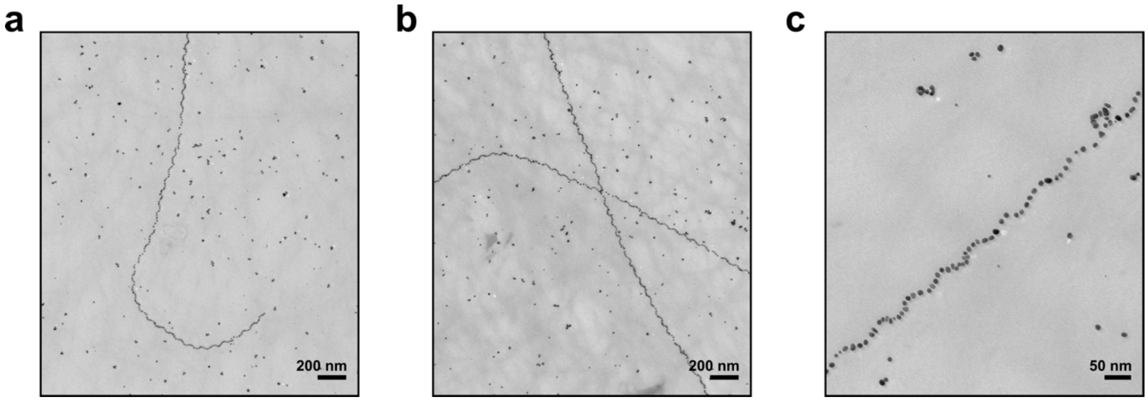

Figure S25. Additional TEM images of AuNP assemblies produced by C_{16}-(AYSSGAPPTPPF)$_2$.

7. Computational Methodology and Supporting Data

General Simulation Set-up: All molecular dynamics (MD) simulations were performed using the GROMACS software package (version 2021).[1] The simulation system comprised one Au slab presenting the (111) surface in an orthorhombic periodic simulation cell with dimensions 5.8nm x 6.1nm x 6.8nm, with the z-axis perpendicular to the Au(111) surface. During all MD simulations, all Au atoms in the slab were held fixed in space during these simulations, with only the Au atom dipoles able to freely rotate. Random initial dipole positions were used throughout. Our previous tests indicated very little difference between binding obtained using a rigid substrate, vs. using a slab where all atoms are free to move.[2] Each simulation comprised one peptide, a slab of five layers of Au atoms, presenting the Au(111) surface on both facets, and approximately 6000 water molecules. Frames were saved every 1 ps unless stated otherwise.

All simulations were performed in the Canonical (NVT) ensemble at 300K, using the Nose-Hoover thermostat.[3,4] The MD leap-frog algorithm[5] was used to integrate Newton's equations of motion using a timestep of 1 fs. The Verlet grid cut-off scheme[6] was applied for neighbor searching. The particle-mesh Ewald (PME) algorithm[7] was used for calculating electrostatic interactions. The CHARMM22* force field[8,9] was used for the peptides, the modified TIP3P potential was used for water,[10] and the polarizable GolP-CHARMM force-field[11] was used for the Au slab.

Replica Exchange with Solute Tempering MD simulations: REST-MD simulations for each of the six peptides ($PEP_{Au}^{A,9}$, $PEP_{Au}^{S,9}$, $PEP_{Au}^{C,9}$, $PEP_{Au}^{T,9}$, $PEP_{Au}^{CtBu,9}$, $PEP_{Au}^{CoxtBu,9}$) were run in the adsorbed state at the aqueous Au(111) interface. Sixteen replicas were used with an 'effective temperature' window of 300-430 K following the Terakawa implementation,[12,13] as reported

previously. Note that the effective temperature is used to determine the scaling factors to modify the Hamiltonian, and does not refer to the thermal temperature of the system (which remained at 300 K throughout). The initial peptide backbone structures of the 16 replicas were taken from previous work,[14] and captured a range of different secondary structures as well as random coil structures. The 16 values of lambda used to scale our force-field were: λ_i = 0.000, 0.057, 0.114, 0.177, 0.240, 0.310, 0.382, 0.458, 0.528, 0.597, 0.692, 0.750, 0.803, 0.855, 0.930, and 1.000, as established in previous work. Prior to each production REST-MD simulation, the 16 initial configurations were energy minimized and then equilibrated at their target potential for 0.5 ns, with no exchange moves attempted during this period. During the production run, exchanges were attempted every 1 ps. Each REST-MD trajectory was of 15 ns duration (amounting to 16× 15 ns = 0.24 μs of nominal total simulation time).

Clustering Analysis: Structures in each production trajectory were classified into groups (herein referred to as clusters) on the basis of similarity in the peptide backbone conformation (backbone defined as 36 atoms per peptide: amide nitrogen, alpha carbon, and carbonyl carbon per each residue), as determined by the root mean square deviation (RMSD) in those backbone atom positions. Clustering was performed over all 15001 frames of each production REST-MD trajectory using the Daura algorithm[15] using the gmx-cluster utility with a cut-off of 2.0Å in the RMSD of selected atomic positions. The number of frames in each cluster is used to determine the relative population of each cluster. Cross-cluster similarity (i.e. determining the backbone conformation similarity between clusters of different peptides) was evaluated based on the RMSD of the backbone atomic positions for the top five most populated clusters for each peptide (based on the cluster centroid structure in each case) in the pair under comparison. A matched pair of

clusters was defined to have an RMSD value smaller than 2.0 Å and a near-matched pair was similarly defined to have an RMSD greater than 2.0 Å and less than or equal to 2.5 Å.

Residue contact analysis: To quantify residue-surface contact for each peptide in the REST-MD simulations, the vertical distance (perpendicular to the surface plane) between the topmost Au atoms on the Au(111) surface and a reference site on each residue (summarized in Table S2) was calculated for every frame in each REST-MD trajectory. The residue was considered as in contact with Au surface if the measured distance was less than or equal to the cut-off values used here (which have been determined and published elsewhere[16]). For the non-standard residues, we used the sulfur atom as the reference site and a cut-off value of 4.5Å for determining the surface contact. The summary of reference sites and cut-off values are shown in Table S2. The degree of residue surface contact is expressed as a percentage of the 15001 frames for which that specific residue was determined to be in contact.

Table S2. Reference sites and cut-off values for each residue used to determine residue-surface contact.

Residue	Reference atom	Cut-off (Å)
Ala	Beta carbon	4.5
Tyr	c.o.m. of ring heavy atoms	4.0
Ser	Side-chain oxygen	4.3
Gly	Alpha carbon	4.6
Pro	Gamma carbon	4.5
Phe	c.o.m. of ring heavy atoms	4.0
Cys	Sulfur	3.5
Met	Sulfur	3.5
Thr	Side-chain oxygen	4.0
M^{ox}	Sulfur	4.5
CtBu	Sulfur	4.5
C^{ox}tBu	Sulfur	4.5

Steered MD and umbrella sampling calculations: The umbrella sampling approach was used to evaluate the potential of mean force profiles of M^{ox}, CtBu and C^{ox}tBu amino acid analogues binding at the aqueous Au(111) interface. The residue binding energy profiles were calculated using similar methodology described in previous work.[17] Both the N- and C-termini of these amino acid were capped. Steered pulling simulations were conducted to accelerate the binding progress along the direction perpendicular to the surface plane (along the z-axis) with a constant speed, thus the harmonic force constant for the steered MD and subsequent umbrella sampling simulations

was set to 3000 kJ mol^{-1} nm^{-2}, with a pulling rate of 0.05 nm ns^{-1}. The spatial interval between adjacent umbrella sampling windows was 0.05 nm along the z-axis (defined as the reaction coordinate), in which each umbrella sampling window was centered at each value of the reaction coordinate, and an *NVT* simulation under the applied force constant was run for 100 ns per window. The resultant PMF profiles with estimated errors were obtained using WHAM using "traj" bootstrapping method with 200 bootstraps and a default tolerance of 10^{-6} using the gmx-wham utility.[18]

Table S3. Amino acid binding free energies in kJ mol^{-1} (as reported in Palafox-Hernandez *et al.*, *Chem. Mater.* 2014, 26, 4960-4969). Values for CtBu, M^{ox} and C^{ox}tBu were obtained in current work.

Amino acid	Binding E.
A	-9.0
Y	-31.5
S	-10.0
G	-15.0
P	-13.0
F	-20.0
C	-32.0
M	-23.0
CtBu	-23.0
Mox	-11.6
T	-12.5
CoxtBu	-12.9

Table S4. Residue surface contact ("Contact") expressed as a fraction between 0 and 1, and residue binding score ("Bind Sc.", kJ mol^{-1}) defined as the contact fraction multiplied by the residue binding free energy (Table S3), for the C, M and CtBu peptide variants. Blue highlighting indicates the 9th residue.

C			M			CtBu		
Residue	Contact	Bind Sc.	Residue	Contact	Bind Sc.	Residue	Contact	Bind Sc.
A	0.08	-0.72	A	0.03	-0.27	A	0.1	-0.9
Y	0.65	-20.48	Y	0.98	-30.87	Y	0.81	-25.52
S	0.35	-3.5	S	0.23	-2.3	S	0.15	-1.5
S	0.52	-5.2	S	0.58	-5.8	S	0.34	-3.4
G	0.52	-7.8	G	0.62	-9.3	G	0.56	-8.4
A	0.77	-6.93	A	0.73	-6.57	A	0.48	-4.32
P	0.41	-5.33	P	0.41	-5.33	P	0.32	-4.16
P	0.12	-1.56	P	0.17	-2.21	P	0.2	-2.6
C	0.88	-28.16	M	0.96	-22.08	CtBu	0.55	-12.65
P	0.27	-3.51	P	0.45	-5.85	P	0.6	-7.8
P	0.05	-0.65	P	0.06	-0.78	P	0.13	-1.69
F	0.76	-15.2	F	0.95	-19	F	0.73	-14.6

Table S5. Residue surface contact ("Contact") expressed as a fraction between 0 and 1, and residue binding score ("Bind Sc.", kJ mol^{-1}) defined as the contact fraction multiplied by the residue binding free energy (Table S3), for M^{ox}, S, and T peptide variants. Blue highlighting indicates the 9th residue.

Mox			S			T		
Residue	Contact	Bind Sc.	Residue	Contact	Bind Sc.	Residue	Contact	Bind Sc.
A	0.06	-0.54	A	0.14	-1.26	A	0.07	-0.63
Y	0.97	-30.56	Y	0.86	-27.09	Y	0.88	-27.72
S	0.23	-2.3	S	0.09	-0.9	S	0.1	-1
S	0.5	-5	S	0.36	-3.6	S	0.21	-2.1
G	0.66	-9.9	G	0.29	-4.35	G	0.23	-3.45
A	0.67	-6.03	A	0.46	-4.14	A	0.15	-1.35
P	0.44	-5.72	P	0.27	-3.51	P	0.17	-2.21
P	0.19	-2.47	P	0.26	-3.38	P	0.4	-5.2
Mox	0.58	-6.73	S	0.63	-6.3	T	0.38	-4.75
P	0.36	-4.68	P	0.25	-3.25	P	0.33	-4.29
P	0.11	-1.43	P	0.05	-0.65	P	0.17	-2.21
F	0.81	-16.2	F	0.81	-16.2	F	0.61	-12.2

Table S6. Residue surface contact ("Contact") expressed as a fraction between 0 and 1, and residue binding score ("Bind Sc.", kJ mol^{-1}) defined as the contact fraction multiplied by the residue binding free energy (Table S3), for A and C^{ox}tBu peptide variants. Blue highlighting indicates the 9th residue.

A			CoxtBu		
Residue	Contact	Bind Sc.	Residue	Contact	Bind Sc.
A	0.02	-0.18	A	0.13	-1.17
Y	0.97	-30.56	Y	0.77	-24.26
S	0.4	-4	S	0.17	-1.7
S	0.63	-6.3	S	0.35	-3.5
G	0.65	-9.75	G	0.38	-5.7
A	0.68	-6.12	A	0.25	-2.25
P	0.51	-6.63	P	0.2	-2.6
P	0.11	-1.43	P	0.43	-5.59
A	0.3	-2.7	CoxtBu	0.13	-1.68
P	0.3	-3.9	P	0.22	-2.86
P	0.07	-0.91	P	0.17	-2.21
F	0.92	-18.4	F	0.73	-14.6

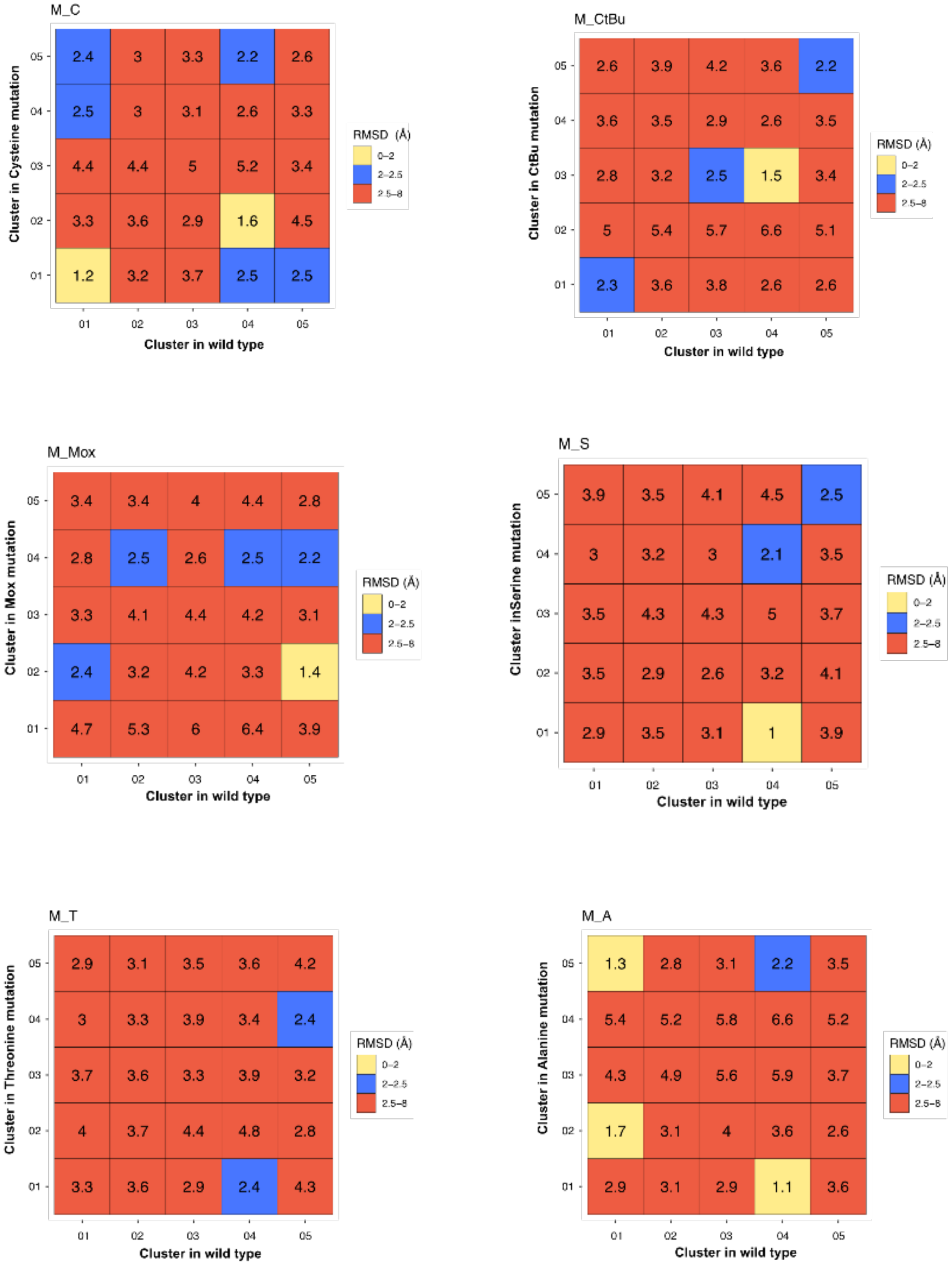

Figure S26. Results from cross-cluster comparison of cluster centroid of the top 5 clusters of the original ("wild type") peptide and those of the peptide variants. Light-colored squares indicate conformational matches and the blue squares indicate near-matched structures.

8. References

(1) Abraham, M. J.; Murtola, T.; Schulz, R.; Páll, S.; Smith, J. C.; Hess, B.; Lindahl, E. GROMACS: High Performance Molecular Simulations through Multi-Level Parallelism from Laptops to Supercomputers. *Softwarex* 2015, *1*, 19–25.

(2) Wright, L. B.; Freeman, C. L.; Walsh, T. R. Benzene Adsorption at the Aqueous (0 1 1) α-Quartz Interface: Is Surface Flexibility Important? *Mol Simulat* 2013, *39*, 1093–1102.

(3) Nosé, S. A Molecular Dynamics Method for Simulations in the Canonical Ensemble. *Mol. Phys.* 1984, *52*, 255–268.

(4) Hoover, W. G. Canonical Dynamics: Equilibrium Phase-Space Distributions. *Phys. Rev. A* 1985, *31*, 1695–1697.

(5) Gunsteren, W. F. V.; Berendsen, H. J. C. A Leap-Frog Algorithm for Stochastic Dynamics. *Mol Simulat.* 1988, *1*, 173–185.

(6) Páll, S.; Hess, B. A Flexible Algorithm for Calculating Pair Interactions on SIMD Architectures. *Comput. Phys. Commun.* 2013, *184*, 2641–2650.

(7) Darden, T.; York, D.; Pedersen, L. Particle Mesh Ewald: An N ·log(N) Method for Ewald Sums in Large Systems. *J Chem. Phys.* 1993, *98*, 10089–10092.

(8) MacKerrell Jr., A. D.; Bashford, D.; Bellott, M.; Jr, R. L. D.; Evanseck, J. D.; Field, M. J.; Fischer, S.; Gao, J.; Guo, H.; Ha, S.; Joseph-McCarthy, D.; Kuchnir, L.; Kuczera, K.; Lau, F. T. K.; Mattos, C.; Michnick, S.; Ngo, T.; Nguyen, D. T.; Prodhom, B.; III, W. E. R.; Roux, B.; Schlenkrich, M.; Smith, J. C.; Stote, R.; Straub, J.; Watanabe, M.; Wiórkiewicz-Kuczera, J.; Yin, D.; Karplus, M. All-Atom Empirical Potential for Molecular Modeling and Dynamics Studies of Proteins†. *J. Phys. Chem. B* 1998, *102*, 3586–3616.

(9) Piana, S.; Lindorff-Larsen, K.; Shaw, D. E. How Robust Are Protein Folding Simulations with Respect to Force Field Parameterization? *Biophys. J.* 2011, *100*, L47–L49.

(10)Jorgensen, W. L.; Chandrasekhar, J.; Madura, J. D.; Impey, R. W.; Klein, M. L. Comparison of Simple Potential Functions for Simulating Liquid Water. *J. Chem. Phys.* 1983, *79*, 926–935.

(11) Wright, L. B.; Rodger, P. M.; Corni, S.; Walsh, T. R. GolP-CHARMM: First-Principles Based Force Fields for the Interaction of Proteins with Au(111) and Au(100). *J. Chem. Theory Comput.* 2013, *9*, 1616–1630.

(12) Terakawa, T.; Kameda, T.; Takada, S. On Easy Implementation of a Variant of the Replica Exchange with Solute Tempering in GROMACS. *J. Comput. Chem.* 2011, *32*, 1228–1234.

(13) Wright L. B.; Walsh, T. R., Efficient conformational sampling of peptides adsorbed onto inorganic surfaces: Insights from a quartz binding peptide*, Phys. Chem. Chem. Phys.* 2013, *15*, 4715-4726.

(14) Mokashi-Punekar, S.; Walsh, T. R.; Rosi, N. L. Tuning the Structure and Chiroptical Properties of Gold Nanoparticle Single Helices via Peptide Sequence Variation. *J Am Chem Soc* 2019, *141*, 15710–15716.

(15) Daura, X.; Gademann, K.; Jaun, B.; Seebach, D.; Gunsteren, W. F. van; Mark, A. E. Peptide Folding: When Simulation Meets Experiment. *Angew. Chem., Int. Ed.* 1999, *38*, 236–240.

(16) Tang, Z.; Palafox-Hernandez, J. P.; Law, W.-C.; Hughes, Z. E.; Swihart, M. T.; Prasad, P. N.; Knecht, M. R.; Walsh, T. R. Biomolecular Recognition Principles for Bionanocombinatorics: An Integrated Approach To Elucidate Enthalpic and Entropic Factors. *ACS Nano* 2013, *7*, 9632–9646.

(17) Jin, R.; Walsh, T. R. Modeling-Led Materials-Binding Peptide Design for Hexagonal Boron Nitride Interfaces. *Adv. Mater. Interfaces* 2022, 2102397.

(18) Hub, J. S.; Groot, B. L. de; Spoel, D. van der. g_wham-A Free Weighted Histogram Analysis Implementation Including Robust Error and Autocorrelation Estimates. *J. Chem. Theory Comput.* 2010, *6*, 3713–3720.
